# Nature-based solutions in spatial planning and policies for climate change adaptation: A literature review

**DOI:** 10.1007/s13280-024-02052-1

**Published:** 2024-07-30

**Authors:** João Corgo, Sara Santos Cruz, Paulo Conceição

**Affiliations:** 1 CITTA – Research Centre for Territory, Transports and Environment, Porto, Portugal; 2 Department of Civil Engineering, Faculty of Engineering, University of Porto, Rua Dr. Roberto Frias S/N, 4200-465 Porto, Portugal

**Keywords:** Climate change adaptation, Nature-based solutions, Policy instruments, Spatial planning

## Abstract

Nature-based solutions (NbSs) are recognised as relevant to spatial planning in addressing societal challenges, although their uptake is limited and fragmented to some case studies, and difficulties emerge from their implementation and operationalisation. The research developed a literature review to investigate how NbS has been considered for its implementation and operationalisation in spatial planning and how NbS has been included across different policy instruments and used to address climate change adaptation (CCA). The results highlighted: Firstly, the review contributed to bridge the gap in NbS implementation and operationalisation by proposing a novel three-dimensional categorisation system to guide the selection of suitable NbS principles to address societal challenges; secondly, this study still revealed gaps in some policy areas, despite the effort to extensively apply NbS across diverse policy instruments to CCA. Overall, the review further emphasises the need for future research focused on monitoring and evaluating NbS's effectiveness to CCA.

**Supplementary Information:**

The online version contains supplementary material available at 10.1007/s13280-024-02052-1.

## Introduction

Urban areas face a range of societal challenges that put humans, nature, and biodiversity under extreme pressure to function and survive (UN 2018). Increasingly, more key biodiversity areas succumb to urbanisation and encroachment, jeopardising ecosystems' vital role in ensuring the health and safety of urban life (Weith et al. 2020). Climate change adaptation (CCA) is imperative in managing these impacts and ensuring the resilience of urban ecosystems. CCA refers to the process of adjusting to the present and future effects of climate change, encompassing measures to reduce vulnerability and enhance the capacity of socio-ecological systems to cope with climate-related challenges (IPCC 2022a). Due to this scenario, the nature-based solutions (NbSs) concept has been increasingly considered as part of the solutions for sustainable urban land management and to increase urban resilience.

Nature has been used as a solution in various contexts for a long time and as a mediator in the relationship between territories and humans for their well-being (Liu et al. 2022). In this regard, the concept of NbS was first coined in the early 2000s to explore innovative approaches for adapting to and mitigating the effects of climate change (CC) while also safeguarding biodiversity and promoting sustainable livelihoods (MacKinnon et al. 2008; Cohen-Shacham et al. 2016; Somarakis et al. 2019) at integrating ecosystem services (ESs) into urban planning (Escobedo et al. 2019; Longato et al. 2023).

Due to its integrated approach, NbSs have been defined and applied in various ways (Appendix S1, Table S1). The two most prominent are from the European Commission (EC) (EC 2015) and the International Union for Conservation of Nature (IUCN) (Cohen-Shacham et al. 2016). In both, these solutions address societal challenges by using ecosystem functions and providing several benefits for nature and people. However, the EC definition employs a broader notion, including actions inspired and supported by nature (EC 2015), while the IUCN focuses more on the protection, sustainable management, and restoration of ecosystems (Cohen-Shacham et al. 2016). The EC's NbS definition sees nature as a driver for innovation, promoting environmental, social, and economic benefits for a sustainable and competitive Europe. This article uses the EC definition of NbS as solutions that are inspired and supported by nature, which are cost-effective, simultaneously provide environmental, social and economic benefits and help build resilience. Such solutions bring more and more diverse, natural features, and processes into cities, landscapes and seascapes through locally adapted, resource-efficient, and systemic interventions (EC 2015, p. 24).

In early 2022, the United Nations Environment Assembly (UNEA) of the United Nations Environment Programme (UNEP) developed a new definition of NbS, recognising the need for a multilaterally and globally accepted definition that aligns with all the ecosystem-based approaches (EbAps) encompassed within the NbS framework (UNEP 2022). This new definition recognises NbS as an essential contribution to climate action, especially for climate change adaptation and mitigation (CCAM) (UNEP 2022), in line with the first EC concept of NbS (EC 2015). The use of NbS has grown over the last decades into a wide concept that encompasses a green, sustainable, and harmonious development and synergises human and environmental outcomes (Cohen-Shacham et al. 2016; EC 2021).

Acknowledging the current global climate crisis, researchers, planners, policymakers, and decision-makers has consensually recognised the relevant role of NbS in addressing different societal challenges. However, there is a pressing need to further understand how NbS works as an ally in tackling the climate crisis. Moreover, spatial planning has been using tools and methodologies to apply NbS to tackle the growing societal challenges that negatively impact nature's capacity to provide resources (Cohen-Shacham et al. 2019; Kabisch et al. 2022).

Despite the extensive number of academic publications regarding NbS (Bayulken et al. 2021), its uptake in planning and practice has been limited and sometimes fragmented to some front-running urban case studies (Grace et al. 2021). This research study recognises the need to understand how NbS and CCA have been mainstreamed across different scales of policies that also help to implement and operationalise the use of NbS in spatial planning practice. Its main objectives are:

- Investigate how NbS has been considered for its implementation and operationalisation in spatial planning, in a theoretical way.
- Explore how NbS and CCA have been included across different policy instruments:
  - Analyse the policy instruments at different areas and scales.
  - Identify if NbS are intended to tackle CCA.

At the same time, it recognises NbS' potential benefits and drawbacks, aiming to answer the proposed objectives.

To investigate the integration of NbS and CCA into policy instruments, an analysis was conducted on various global, European, and national policy instruments. In order to analyse national policy instruments, the Portuguese context was considered. The research is justified by some reasons: (a) CC has global impacts, requiring policies capable of responding to these changes within international communities (Castellari et al. 2021; IPCC 2022b); (b) CC significantly affects both the European Union and Portugal, underscoring the need to formulate and implement climate adaptation strategies (AdaPT.Local, 2018; IPCC 2022b); (c) the EU has been actively designing and implementing NbS projects in its urban areas, with Portugal serving as a notable example (Somarakis et al. 2019), and (d) recently, the EU has undertaken numerous projects and research initiatives focusing on NbS and related approaches (e.g., BiodivERsA, EKLIPSENature4Cities, NATURVATION, THINK NATURE, and LIFE-myBUILDINGisGREEN).

At the national level, Portugal was considered as a case study to investigate the integration of NbS and CCA in policy instruments due to the following specific reasons:

- Similar to other EU member states, Portugal has aligned its policies with EU directives and initiatives on NbS and CCA. This alignment provides valuable insights into how EU-level objectives are translated and implemented at the national level.
- While examples of NbS case studies exist in Portugal, we aim to understand if the concept is formally integrated into planning instruments and considered for climate adaptation.

Acknowledging the need for transformative solutions in the face of high-end climate change, conventional adaptation strategies prove insufficient in averting major disruptions in social–ecological systems (Tàbara et al. 2019). In this context, NbSs emerge as capable solutions to ensure the transformative capacity of socio-ecological systems to address various societal challenges (Scolobig et al. 2023). ‘Transformative capacity’ can be defined as the capacity that supports transformative strategies that aim to facilitate ‘*adjusting to the new impacts of climate change*’ and ‘*creating a new system’* when the existing system is no longer tenable or desirable (Mehryar et al. 2022, p. 3). Transformative approaches are essential for NbS to adapt effectively to climate change and enable societies to cope and adjust to its impacts (Mehryar et al. 2022). Transformation is fundamental when adaptation goes beyond the limits of a system (Manyena et al. 2019; Mehryar et al. 2022). The distinction between adaptation and transformation relies on the degree of change, with transformation turning clearer when the system is changed or dismantled to create a new system (Mehryar et al. 2022). Thus, when NbS projects cannot adapt to the challenge, their ecosystems must be proactive and transform themselves to overcome it (Mehryar et al. 2022).

The article is divided into five parts: The methodology in part 2 is based on a literature review regarding three different review methods that complement each other, resulting in a comprehensive literature review. Part 3 provides the results regarding the consideration of NbS for its implementation and operationalisation in spatial planning practice and a review concerning NbS and CCA in policy instruments. Part 4 discusses the results, and finally, part 5 provides the conclusions.

## Methodology

To fulfil the objectives, the study undertook a comprehensive and articulated literature review based on three different but complementary literature review methods. A semi-systematic method (SSM) was following the PRISMA 2020 methodology to comprehensively explore current knowledge on NbS and other related topics. To complement the structured search of the SSM and capture a wider range of potentially relevant studies, a narrative or traditional method (NTM) was applied, focusing on key themes and works in the field. Finally, a snowballing method (SM) was used to identify additional studies by examining the reference lists of retrieved articles from both the SSM and NTM. A data analysis was also conducted aiming to synthesise, understand, and interpret the information gathered from all the sources collected.

### Data collection

#### Semi-systematic method (SSM)

**Fig. 1:**
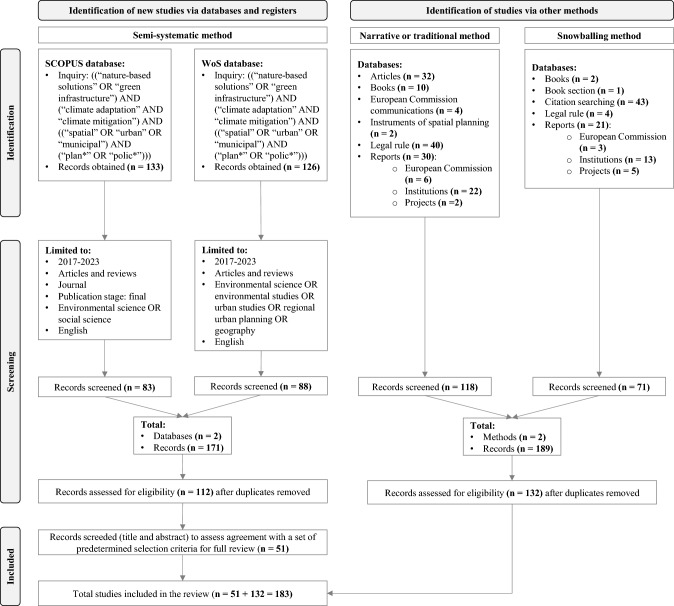
Overview of the literature review process. Based on the PRISMA 2020 flow diagram for systematic reviews

Following Page et al. (2021), the literature review based on the SSM was developed partially according to the PRISMA 2020 methodology. This review was guided by different keywords followed by the analyses of the obtained documents from the NTM aiming to identify research gaps and opportunities. A SSM approach was used, since it is designed for topics that are being conceptualised differently and studied by different groups of researchers from multiple disciplines and that makes it challenging to perform a full systematic review process (Wong et al. 2013). It is then possible to understand how research within the chosen topics has progressed over time and across different research areas. This study checked several of the 27 items (e.g., title, abstract, introduction, methods, results, discussion, and other information) from the PRISMA 2020 statement to guide the literature review. The literature search was also organised according to the PRISMA 2020 flow diagram (Fig. 1) used to resume the identification of studies via databases according to three steps: identification of records from databases; records screening; and studies included in review. The search string employed was: ((‘nature-based solutions' OR ‘green infrastructure') AND (‘climate adaptation' AND ‘climate mitigation') AND ((‘spatial' OR ‘urban' OR ‘municipal') AND (‘plan*' OR ‘polic*'))).

The first step (identification) started with selecting keywords for inquiry, identifying records from the Web of Science (WoS) and Scopus databases. A group of keywords was selected to perform the literature search on WoS and Scopus based on an initial NTM that allowed to obtained global and European reference documents regarding the topic of NbS, such as Cohen-Shacham et al. (2016), EC (2015), and UNEP (2022). In line with objectives, the keywords were ‘nature-based solutions’ and ‘green infrastructure’. The words ‘climate adaptation’ and ‘climate mitigation’ were selected regarding the topic of ‘climate change’. The keywords ‘spatial’, ‘urban’, and ‘municipal’ were chosen for the spatial planning area at the municipal level, and the two keywords ‘plan*’ and ‘polic*’ for the planning and policy dimensions. The literature search combined all keywords using the Boolean operators ‘AND’, ‘OR’, and ‘*’. At the end of the first step, a total of 259 records were retrieved, 133 from Scopus and 126 from WoS.

In the second step (screening), the literature search was limited to a list of ‘articles’ and ‘reviews’ published in ‘English’ between ‘2017’ and ‘2023’ to collect the most recent publications about those topics. Following the initial definitions established by EU in 2015 and the IUCN in 2016, the field of NbS has witnessed significant advancements and knowledge accumulation. To capture these latest developments and ensure a manageable review process, this study focused on publications from 2017 to 2023. Besides, different science categories were chosen to fully embrace topics from NbS and CCA studies, this screening phase resulted in 171 records, 83 from Scopus, and 88 from WoS. Followed by the removal of duplicate records, 112 records were listed for analysis.

The last step (included) considered a list of criteria (Table 1) for selecting the final records to review. The list of yes/no questions focused on evidence-based articles related to NbS, particularly GI, in the context of addressing climate change through spatial planning and urban policies at the municipal or urban level. Each of the 112 records obtained from the screening step was screened by title and abstract regarding the four main criteria. Each criterion was applied by order, and documents that did not acknowledge with one criterion were excluded. A total of 51 documents complied with the selection criteria and were thoroughly reviewed to identify the knowledge gaps according to the defined objectives.

**Table 1:** List of criteria used to select documents for further review by application order

Criteria for the literature search
Does the article focus on nature-based solutions or green infrastructure? (yes/no)
Does the article address climate change mitigation and/or adaptation? (yes/no)
Does the article address issues relate to spatial planning and/or urban policies? (yes/no)
Does the article work on a municipal or urban scale? (yes/no)

#### Narrative or traditional method (NTM)

The primary purpose of the narrative or traditional method (NTM) was to explore and present a comprehensive understanding of the different topics explored (Hart 1998). It also supported the SSM since it aimed to synthesise and summarise information from various sources in a narrative or descriptive manner, offering a broader understanding of the studied subjects (Green et al. 2006; Petticrew and Roberts 2006). Unlike the SSM, the NTM provided flexibility in selecting and interpreting the literature allowing a more subjective analysis and interpretation of the information, which led to valuable insights that might not have been captured by the SSM alone. The NTM employed a broad search strategy, identifying a wide range of total 118 sources, such as academic articles, books, some spatial planning instruments, several global, EU, and Portuguese legal rules, and different reports from distinct sources, such as the European Commission, other institutions, and projects. The selection process considered factors like source credibility, publication date, and relevance to the research question. The NTM indicated the need to develop research regarding the implementation and operationalisation of NbS in spatial planning practice, as well as explore how NbS and CCA have been included in different policy instruments and understand if NbSs are used to tackle CCA.

Nonetheless, NTM can have limitations by relying on the expertise and interpretation of the researcher, which can introduce bias (Petticrew and Roberts 2006). Their lack of a systematic and replicable methodology may also decrease the transparency and reliability of the review process (Cooper 1988). To overcome these limitations, the study adopted the presented semi-systematic review based on clear objectives and the criteria.

#### Snowballing method (SM)

Aiming to collect more inside, explore and analyse the information obtained from the 51 articles of the SSM, a snowballing method (SM) was applied. SM is a widely used research method that identify additional relevant sources from existing references. Originally introduced by Coleman (1958) in sociological research, the snowballing method has gained significant attention since it allows identifying important references and captures relevant studies, not possible through traditional database searches (Wohlin 2014). It enabled identifying additional pertinent articles by following citation chains and reference lists of the initially retrieved articles. The method followed two different directions: backward and forward. Backward snowballing involved examining the references cited within those articles, while forward snowballing involved tracking forward citations of the initial articles (Wohlin 2014). Both directions were combined to maximise the identification of relevant studies regarding the topics being studied—that might not be indexed in the Scopus and WoS databases. This method can also present some limitations as the risk of a bias due to selective inclusion of studies and/or missing articles if the initial set is not comprehensive (Featherstone et al. 2015). The risk was minimised using the comprehensive set of articles obtained from SSM.

In total, 71 new references were compiled, such as books and book sections, a new group of articles, regulations and reports from the EC, and other international institutions and projects.

### Data analysis

Given the 183 included documents, a comprehensive literature review explored the multifaceted aspects of NbS as a concept in the context of spatial planning and policy for CCA and delved into three key topics:

- NbS umbrella concept
- NbS implementation and operationalisation in spatial planning
- NbS and CCA in policy instruments

Content and descriptive analysis were used to analyse the NbS umbrella concept. The content analysis determined the various EbAp encompassed by the NbS concept and grouped them into distinct categories based on their commonalities, according to Cohen-Shacham et al. (2016, 2019) and the European Commission (2021). Then, the descriptive analysis allowed the clear summarisation and organisation of the identified categories, outlined distinct categories, further enriched by providing specific examples.

Then, the research then explored different NbS principles frameworks (IUCN 2016) that have been recognised as potential guides to ensure and facilitate NbS implementation and operationalisation in spatial planning. An interpretive thematic analysis was developed according to their objectives and scope to gain a further understanding of these principles.

Lastly, a content analysis explored how NbS are integrated into policy documents across different scales (e.g. global, European Union, and Portuguese levels) and sectors. Content analysis assessed both explicit and implicit references to NbS, including related EbAp and concepts and CCA within each document, categorising policies by sector (e.g. climate and urban). This analysis revealed not only the prevalence of NbS within policy instruments and whether these instruments acknowledged NbS as a tool for addressing CCA.

## Results

### The framework of the NbS umbrella concept

**Fig. 2:**
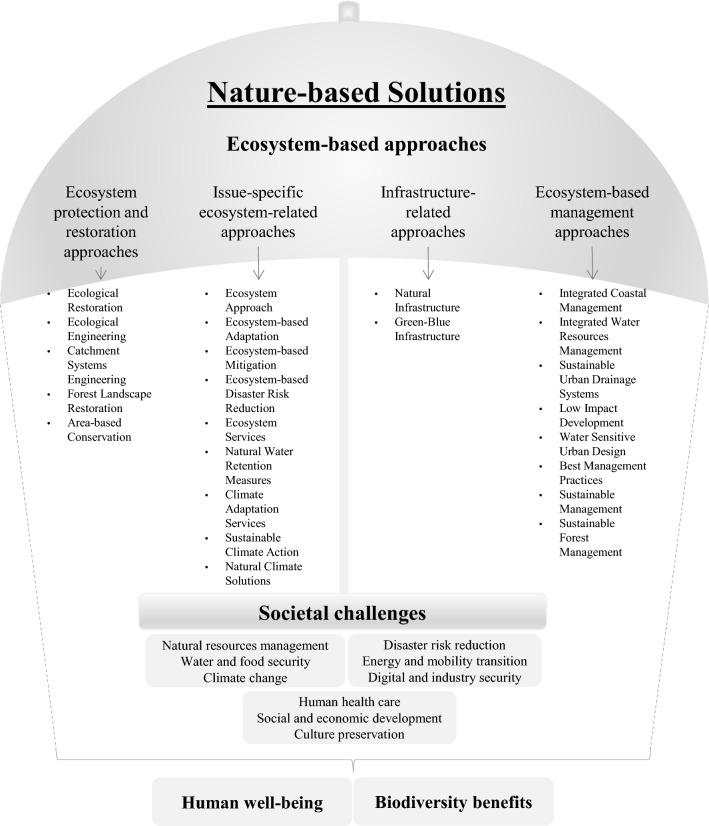
NbS umbrella concept. Adapted from Cohen-Shacham et al. (2016, 2019), European Commission (2021)

The design of solutions to address societal challenges using ecosystem processes is rooted in similar ecosystem approaches (Albert et al. 2019). Policymakers, scientists, and practitioners have been seeking ways to address these challenges from both the institutional and the academic fields, bringing new perspectives to natural environment management through innovative ideas, terminologies, and concepts of the ecosystem approach. NbS is a collective term for innovative solutions based on natural processes and ecosystems to fix those challenges (Ruangpan et al. 2020) and is an umbrella concept since it covers a range of different EbaP and linked concepts (Cohen-Shacham et al. 2016, 2019) (Fig. 2 and Appendix S2, Table S2).

Regarding their scope of application, the NbS EbAp can be organised according to their category type (e.g. ecosystem protection and restoration approaches). The ecosystem protection and restoration category refer to approaches related to the ecosystem’s protection (e.g. protected area management) and recovery. The ecosystem-related category focuses on a specific issue related to ecosystem adaptation and mitigation to some challenge or problem (e.g. flood risk reduction or climate change) and contains three approaches related to climate change. ‘Sustainable climate action’ and ‘nature climate solutions’ emerged recently in the literature and policy documents but focused essentially on climate change mitigation. Furthermore, ‘climate adaptation services’ complements the ‘ecosystem-based adaptation’ approach by developing solutions for CCA. This category of NbS approaches also includes the ecosystem services (ESs) approach because it considers their management and the importance of regulating ecosystems. The third category covers the natural and built infrastructures responsible for creating interconnected green and blue spaces networks that aim to plan and manage natural resources that benefit humans and nature. The last category includes NbS approaches responsible for ecosystem management, especially those related to coastal and water resources management, urban water drainage systems, and urban water management. The diversity of ecosystem-based approaches is responsible for addressing specific or multiple societal challenges (e.g. water and food security, disaster risk reduction, and/or climate change) and simultaneously providing human well-being and biodiversity benefits (Castellari et al. 2021; Cohen-Shacham et al. 2019).

Concepts such as ‘sustainable urban drainage systems’ or ‘water sensitive urban’ address stormwater and water pollution management. The ‘natural infrastructure’ and ‘green–blue infrastructure’ approaches focus on multi-functional natural, semi-natural, and man-made infrastructures that apply natural alternatives as solutions for a specific activity (e.g. urban planning) (Nesshöver et al. 2017). ‘Ecosystem-based adaptation’ aims the conservation of biodiversity, ecosystem services, and climate change, while ‘ecosystem-based disaster risk reduction’ is more related to the immediate and medium-term impacts from the risk of weather, climate, and non-climate-related hazards (Ruangpan et al. 2020).

Due to its variety of EbAp, the concept of the NbS is open to cover different interpretations, which helps encourage stakeholders to participate in its discussion and implementation (Ruangpan et al. 2020). Moreover, NbS provides an opportunity to work and improve existing gray infrastructures, as the traditional engineering solutions or hybrid solutions that are sometimes the only way to produce more effective results, especially when their co-benefits are considered (Alves et al. 2019).

Despite all multiple and interrelated benefits and opportunities, NbS can also entail disadvantages, such as ecosystem disservices (e.g. decrease in soil and water quality and quantity) (Thorn et al. 2021; Wu et al. 2021), green gentrification or social segregation, resulting in the increase of privileged residents caused by the development of private capital (Anguelovski et al. 2018, 2019). Therefore, it is important to beware of those possible negative impacts and anticipate them during the design, implementation, and management of NbS in urban territories.

The review regarding the concept of NbS highlighted its strength. The comprehensive framework encompassed NbS's diverse ecosystem-based approaches. All these integrated approaches can empower spatial planning to address a wide range of societal challenges effectively.

### NbS implementation and operationalisation in spatial planning

Spatial planning involves the strategic coordination of land utilisation, spatial configurations, resource allocation, and economic and social dynamics orchestration. It has endeavoured to strategically steer social, economic, and environmental transformation processes towards specific goals (Huxley and Inch 2020). While the importance of nature's benefits is consensual, the rise in human population and urban development has strained ecosystems' ability to provide services and tackle challenges like biodiversity loss and climate change.

Despite diverse publications on NbS (Bayulken et al. 2021), its theoretical and practical implementation in planning remains constrained and often confined to a few pioneering case study cities (Grace et al. 2021). Scholars have identified and discussed various barriers to NbS uptake (Sarabi et al. 2020), with some questioning the concept itself and its potential impact on urban planning, urban design, and governance (Albert et al. 2019; Escobedo et al. 2019; Mell et al. 2023; Tsatsou et al. 2023). Additionally, concerns have been raised about the scarcity of qualified NbS implementation cases, their measurable outcomes (Grace et al. 2021), institutional obstacles, path-dependencies within urban systems, and a lack of change drivers (Davies and Lafortezza 2019). Other barriers regard the lack of political will and long-term commitment (Hawxwell et al. 2019) or their lack of sense of urgency regarding the use of NbS (Trell and van Geet 2019). NbS development is also often hindered by the community´s unfamiliarity with and unfavourable views towards NbS, contributing to a lack of awareness (Wamsler et al. 2020).

**Fig. 3:**
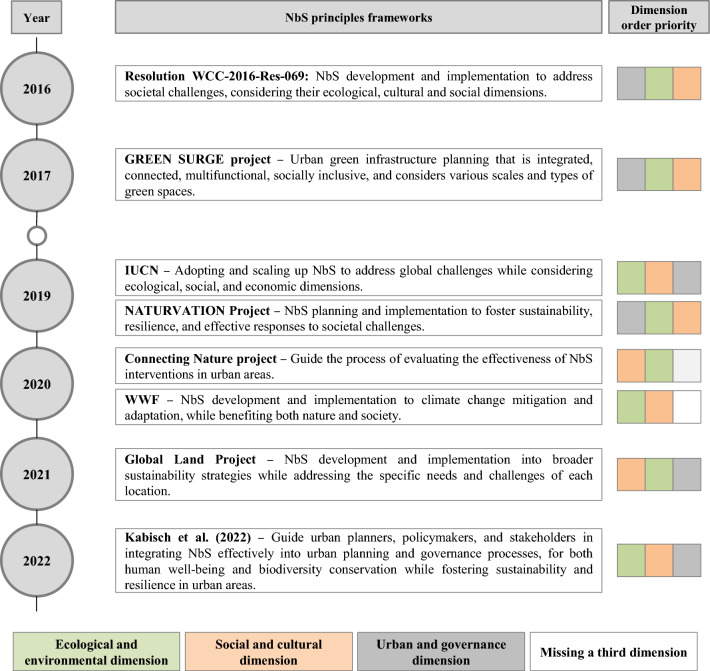
NbS principle frameworks and their corresponding scope for NbS implementation and operationalisation. Each square's colour shows its priority within the 'principle framework': the first square is the top priority, the next is secondary, and the last is the least. A blank square means that there is no third dimension

Due to its overarching goal to address several issues, some authors and documents have presented frameworks of principles that helped clarify the NbS umbrella concept (Cohen-Shacham et al. 2016; EC 2021) and enabled it to be implemented and operationalised. Building on existing principles, they were established to promote the successful implementation and scaling up of NbS (Appendix S3, Table S3—Before IUCN principles). Those principles were an initial attempt to guide what type of interventions should be or could not be considered NbS (IUCN 2012; Eggermont et al. 2015) and its benefits when compared to grey solutions (Anderson et al. 2022). The fundamental principles (Fig. 3) for developing NbS proposals are based on the original NbS principles outlined in the IUCN Programme 2013–2016 (IUCN 2012) and reiterated in the Resolution WWC-2016-Res-069 (IUCN 2016). Recognising their potential, an analysis was conducted wherein each principle framework was described according to its objectives and scope (Appendix S3, Table S3).

Among all the set of principles analysed, it is important to highlight the IUCN's eight principles (IUCN 2016; Cohen-Shacham et al. 2019), the five principles from the Global and Land Project (GLP) (Albert et al. 2021), and the five principles proposed by Kabisch et al. (2022). These principles have implications for NbS planning and implementing and enable a common language between stakeholders (Appendix S3, Table S3—After IUCN principles).

The IUCN principle framework encompasses nature conservation norms and integration with other solutions for global challenges and diverse ES. It considers natural and cultural contexts, promoting equitable societal benefits, supporting biological and cultural diversity and their resilience. The framework is applied at the landscape scale, considering consequences and potential for upscaling, while addressing trade-offs between immediate economic gains and long-term ES. It also promotes the incorporation of NbS into policies to address specific challenges effectively (Cohen-Shacham et al. 2019).

Additionally, the GLP framework developed by Albert et al. (2021) aims to include place-specificity, evidence base, equity, integration, and transdisciplinary into NbS development at landscape scale. As identified by Albert et al. (2021), successful NbS planning should rest on five key principles. These principles not only enhance NbS implementation but also optimize project impact by addressing societal challenges, conserving nature, and delivering ES, all while ensuring community and environmental well-being.

The work of Sowińska-Świerkosz et al. (2023) resonates with the practical application of the IUCN principles framework and the GLP framework, as highlighted by Cohen-Shacham et al. (2019) and (Albert et al. 2021), emphasising the importance of working at the landscape scale for effective NbS implementation. By considering natural and cultural contexts, promoting equity, and addressing trade-offs, these frameworks align with the Sowińska-Świerkosz et al. (2023), emphasising the interconnectedness of NbS and landscape considerations in addressing global challenges and enhancing ecosystem services.

Despite the Cohen-Shacham et al. (2019) and Albert et al. (2021) principles, Kabisch et al. (2022), on the other hand, bring a new perspective that provides a spatial dimension on participatory planning and good governance of NbS to the urban scale. Effectively implementing NbS within cities necessitates a careful reconsideration of their ecological concepts due to urban distinct characteristics. (Beichler et al. 2017; Conway et al. 2019).

To better understand the core of these NbS principle frameworks, they were categorised into different dimensions based on the scope of their principles (Fig. 3), which facilitates their application within the context of implementing NbS interventions to address various societal challenges. Three dimensions were identified—‘ecological and environmental’, ‘social and cultural’, and ‘urban and governance’. The first emphasises the importance of ensuring that NbSs are in harmony with the natural environment and ecosystems, promoting ecological balance, striving for fair and sustainable solutions over the long term, and benefiting both nature and human well-being. It also upholds principles of conservation while ensuring that NbSs are equitable and accessible to all. The second emphasises fairness and long-term sustainability while also extending to human communities' well-being. It also ensures that NbSs are inclusive and beneficial do all members of society. The frameworks of this dimension should recognise the cultural diversity and heritage of communities and how NbS can align with and respect these cultural values. The third highlights the role of urban planners and policymakers in integrating NbS into urban environments. It underscores the importance of systematic evaluations to assess the effectiveness of NbS initiatives in urban settings and recognises that NbSs in urban areas need to be tailored to the specific context and challenges of each location. Each group reflects the primary dimension that each framework predominantly addresses. It is important to note that these dimensions are not mutually exclusive, and many NbS principle frameworks consider multiple dimensions simultaneously. These dimensions collectively provide a comprehensive approach to developing and implementing NbS solutions that consider ecological, social, cultural, urban, and governance factors (Appendix S3, Table S3). An interpretive thematic analysis was conducted to understand the objectives and scopes of these principle frameworks. This analysis focused on identifying recurring themes, resulting in the emergence of three key dimensions. These dimensions capture the range of considerations addressed by the NbS principles frameworks.

To ensure that NbS is well understood, communicated, and implemented, IUCN developed the ‘Global Standard’ (GS) to operationalise its eight NbS principles (Cohen-Shacham et al. 2016, 2019; IUCN 2016) and achieve NbS goals. This GS aims to ensure, through its 28 indicators, the design and implementation of NbS, creating a common understanding of the concept, coordinating with affected sectors, ensuring quality control in design and execution, applying relevant tools and methods, assessing risks of unsustainable nature use, and engaging multiple sectors to address societal challenges (IUCN 2020a, 2020b).

**Fig. 4:**
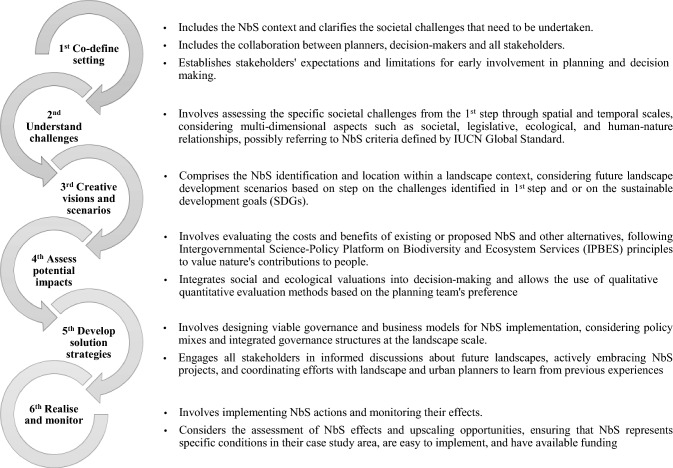
Steps for planning NBS. *Source*: Pascual et al. (2017), Nesshöver et al. (2017), Raymond et al. (2017b), Gulsrud et al. (2018), Dorst et al. (2019), Albert et al. (2019, 2021), Longato et al. (2023)

Despite the existence of various NbS principles frameworks, global standard criteria, and indicators, the research community still emphasise the need for further research into methods that can better integrate of NbS into planning. This is driven by the crucial role of planning in identifying, designing, and implementing NbS (Raymond et al. 2017a; Frantzeskaki 2019). In this context, Albert et al. (2021) identified six steps for a comprehensive approach to planning NbS. These steps were comprised according to an adaptive planning cycle based on the works done by Kato and Ahern (2008) and Ahern et al. (2014) (Fig. 4).

This cycle of steps addresses specific societal challenges rather than just creating comprehensive plans. It takes a multidimensional assessment of the issues at stake, such as the societal, legislative, and ecological dimensions, and the human-nature relationships. It aims to develop practical and actionable strategies as a part of the planning process. Implementing these steps would require increased interdisciplinary collaboration within the planning team, involving planners, ecologists, and social scientists to understand the interactions between human–environment systems better and incorporate this knowledge into the planning process (Albert et al. 2021).

Moreover, Istrate and Hamel (2023) have introduced a conceptual framework to assess various game-based approaches designed to enhance NbS adoption by educating urban stakeholders and actively involving them in the NbS planning process. It aims to evaluate the primary objectives of different games, such as educational, intervention-oriented, or research-focused, and their capacity to encompass key aspects of NbS and urban planning. The authors consider that there is still room for improvement and emphasise the need to develop more context-specific games, especially those tailored to local needs.

Despite all the principles and steps presented, some authors and institutions recognise the ongoing need to apply and tailor these principles to different case studies to better understand their outcomes (IUCN 2020a; Albert et al. 2021). Regardless of some good examples of NbS implementation (Somarakis et al. (2019), the continuous monitoring and evaluation of their progress and functioning remain essential. It is important to acknowledge that ecosystems are dynamic and uncertain, so it is important to recognise that the design objectives may not be achieved entirely. Therefore, monitoring and evaluating the NbS´ effectiveness are a critical step in the planning process (Almassy et al. 2018; Somarakis et al. 2019; EC 2020b; Albert et al. 2021).

In this respect, the literature review emphasised the importance of policy in providing insights into the instruments that may influence the monitoring and evaluation of NbS for its effectiveness in addressing CCA. Although the Green Surge Project tried to understand the planning and governance of urban green infrastructure in Lisbon (Santos et al. 2015a) and Almada (Santos et al. 2015b), both cases in Portugal, the project has not applied NbS principle framework. For this reason, the present investigation reviewed different Portuguese policy documents to investigate if NbS has been integrated into those policies to facilitate its implementation and operationalisation into spatial planning for addressing CCA.

### NbS and CCA in policy instruments

The literature review indicates that global, EU, and Portuguese policies on different areas have progressively embedded NbS for CCA over their objectives, actions, and instruments (Davis et al. 2018; Knoblauch et al. 2019; PNPOT 2019). In the last decades, some policies have been recognising the role of NbS and their ES to protect society and adapt their territories to climate change impacts (PNPOT 2019; Castellari et al. 2021; Manes et al. 2022).

IPBES (2019) and (IPCC 2022b) recognise that the climate and biodiversity crises are interdependent since they share multiple drives, and thus, they should be addressed in unison (Seddon et al. 2019). The EC also recognises this unison in its European Green Deal and its associated strategies (Castellari et al. 2021).

Several policy documents have been addressing the concept of NbS, their EbAp, and related concepts, and promoting synergies for implementing, operationalising, and mainstreaming NbS for CCA. The relevant policies that enhance the implementation of NbS and their related concepts and approaches for CCA are presented in Appendix S4, Table S4 (Davis et al. 2018; Knoblauch et al. 2019; Castellari et al. 2021). The content of each policy presented in the table was analysed and screened according to its explicit or implicit inclusion of EbAp from the NbS umbrella concept (Cohen-Shacham et al. 2016) and its references to CCA.

The policies includes the most relevant instruments, such as regulations, strategies, action plans, agendas, resolutions, or frameworks that are used as the basis for making decisions in politics regarding the implementation and/or degree of support for NbS for CCA. For this purpose, 44 policy agreements (nine global, 19 EU, and 16 national) were analysed. They were organised according to their policy area (e.g. biodiversity and forestry; water and agriculture; maritime; climate; urban; and other cross-cutting policies) following the structure used by Castellari et al. (2021). Despite using different key terms, they provide explicit support for NbS and CCA.

At the global and EU level, the review identified a strong relationship between NbS and CCA, and the strong support and value of the NbS for CCA (Castellari et al. 2021). This synergy was referred in several of the global and EU documents mentioned in Appendix S4, Table S4, namely the ‘United Nations Framework Convention on Climate Change’, ‘Paris Agreement’, ‘Sendai Framework for Disaster Risk Reduction’, ‘2030 Agenda for Sustainable Development’, ‘EU Strategy on Adaptation to Climate Change’, and the ‘European Green Deal’ (Seddon et al. 2019, 2020). Despite these policies consider NbS to adapt territories to CC, their level and nature of support vary considerably in practice (Castellari et al. 2021). Policy frameworks at both the global and EU levels suffer from a lack of coherence (Somarakis et al. 2019) and fragmented governance arrangements (Trémolet 2019). These aspects can hinder effective collaboration, synergies, and joint financing across multiple policy agendas (Castellari et al. 2021). Somarakis et al. (2019) and Castellari et al. (2021) also noticed an important gap at the policy practice level, mainly the lack of using indicators to monitor and evaluate the progress of NbS and its effectiveness to address CCA across this policy arenas.

At the Portuguese level, the policies were chosen according to their relationship to the EU policies and their corresponding transcription from them. The reviewed policies revealed different explicit supports for NbS and CCA. When it comes to climate policies, they explicitly demonstrate strong support for NbS and their associated terms and approaches, recognising their proven value for CCA. For example, the ‘National Strategy for Climate Change Adaptation’, extended until 2025, acknowledges forests as critical ecosystems to provide different ES, with utmost importance to the economy, society, and the environment. This strategy aims to implement NbS in line with CCA and incorporate them into sectoral policies. The national climate law, from 2021, stands as an important milestone within the Portuguese legislative framework, especially amidst the current climate emergency faced by the country, which is expected to worsen (IPCC 2022b). The climate law aims to protect and enhance the regeneration of biodiversity, ecosystems, and their services. It also recognises the vital role of forests and green spaces in adapting rural and urban areas to CC. These areas provide essential ES, such as carbon fixation, habitat formation, and water erosion prevention. Another important component regarding Portugal´s climate change adaptation efforts is the establishment of the ‘Network of Municipalities for Local Adaptation do Climate Change’ (2016), following the ClimAdaPT.Local project promoted by the Portuguese Environment Agency. Recognising climate impacts for local communities, this network aims to boost adaptation at the local level. Several municipalities are called to elaborate their local strategies to increase the capacity of Portuguese municipalities, public and private entities to incorporate CCA in its action policies, planning instruments, and in its interventions (AdaPT.Local 2018). The ‘National Program for Spatial Planning Policy’ (NPSPP), as the main policy for the territorial management in Portugal, represents a key instrument to support NbS for CCA. According to this policy, the development of NbS offers various opportunities within several sectors of spatial planning (e.g. sustainable mobility, circular economy, climate adaptation, and/or regional and municipal ecological structures). The NPSPP underscores the need to align different Portuguese environmental and climate policies to effectively achieve CCA through NbS. This policy is an opportunity to strategically consolidate the national ecological connectivity network within the territory, in line with the principles of a GI, embodying the continuum of ecosystems essential to delineate the MES (PNPOT 2019). In this regard, it may be important to consider the development of a management plan for MES/GI aiming to improve its performance, functions, and the delivery of ES. This plan may function as a tool to connect the various uses and activities within MES/GI, allowing its implementation and management promoting more sustainable territories (Corgo 2021).

## Discussion

Recognising NbS as a collective concept that brings different EbAp according to the societal challenge and acknowledging its capacity to provide multiple and interrelated environmental, social, and economic benefits, opportunities, and some disadvantages, this study addressed its objectives proposing (1) to explore the implementation and operationalisation of NbS in spatial planning and (2) to examine the mainstreaming of NbS and CCA within various policy instruments.

### NbS umbrella concept

The study explored the multifaceted essence of the NbS umbrella concept. While rooted in established ecosystem approaches, NbS emerges as a comprehensive concept encompassing a diverse range of approaches (Albert et al. 2019). This variety strengthens NbS as a powerful tool for spatial planning, allowing for targeted interventions that address a multitude of societal challenges (Ruangpan et al. 2020). The categorisation of NbS approaches, including protection and restoration, issue-specific solutions, infrastructure development, and ecosystem-based management, highlights its adaptability (e.g. green–blue infrastructure or ecosystem-based disaster risk reduction). Furthermore, the inclusion of climate change-focused approaches like ‘ecosystem-based adaptation’ and ‘nature climate solutions’ demonstrates NbS's growing role in tackling this critical global issue. However, the open-ended nature of the NbS concept can lead to varying interpretations among stakeholders (Ruangpan et al. 2020), thus requiring effective communication and collaboration to ensure successful NbS implementation. Additionally, while NbS often complements existing infrastructure, the possibility that traditional engineering solutions or hybrid approaches might be necessary in certain situations should not be overlooked (Alves et al. 2019).

This way, further research is needed to explore how best to prioritise specific NbS approaches within a particular context. Different stakeholders, local communities, practitioners, and policymakers can provide valuable insights into local priorities and potential social impacts of different NbS approaches. Besides, the identification of potential trade-offs and unintended consequences (e.g. ecosystem disservices or social issues like green gentrification) is essential, although they are not within the scope of this paper. Monitoring the effectiveness of the chosen approach and its associated trade-offs allows for adjustments and additional measures if needed (Calliari et al. 2019; European Commission 2021). The NbS umbrella concept needs to be continually updated to incorporate new EbAp as they are developed.

### NbS in spatial planning

The comprehensive literature review in ‘Results' section identified key aspects including challenges associated with NbS implementation, the development of guiding principles to facilitate its implementation and operationalisation, a comprehensive approach to planning NbS, the role of institutions and policies, and the need for ongoing monitoring and evaluation of NbS projects.

The research acknowledged the rich potential of NbS in addressing societal challenges such as biodiversity loss or climate change, but also highlighted the practical challenges in implementing these solutions in spatial planning. These include not only technical challenges like lack of qualified implementation cases and measurable outcomes but also institutional barriers, political will, and community awareness regarding NbS potential. The lack of a clear understanding of NbS benefits contributes to the hesitancy in their adoption (Albert et al. 2019; Escobedo et al. 2019; Sarabi et al. 2020; Grace et al. 2021). This sets the stage for the investigation of how NbSs have been theoretical integrated and operationalised in spatial planning.

To guide those processes, various NbS principle frameworks have been proposed, namely the IUCN principles, the GLP, and those by Kabisch et al. (2022). Overall, they offer guidance on the principles that should underpin NbS planning, emphasising factors like equity, integration, and evidence-based decision-making, establishing a common language for stakeholders and fostering effective collaboration (Cohen-Shacham et al. 2019).

To highlight the value added by these NbS principle frameworks, three different dimensions were proposed: ‘ecological and environmental’, ‘social and cultural’, and ‘urban and governance’ (Fig. 3). The purpose was to categorise these frameworks based on their principles, enhancing their relevance in implementing NbS interventions. Noteworthy, these dimensions are not mutually exclusive; indeed, many NbS principle frameworks encompass multiple dimensions simultaneously. Collectively, they offer a comprehensive approach, supporting the selection of the appropriate framework for addressing specific societal challenges.

To guarantee a comprehensive grasp, effective communication, and successful implementation of NbS, the IUCN developed its Global Standard (IUCN 2020a, 2020b). This standard, backed by a set of indicators, aims to ensure that NbS projects follow the defined principles. The GS underscores the importance of coordination, quality control, risk assessment, and sector engagement in NbS implementation. This operationalisation is vital to ensure that NbS truly deliver the intended benefits and address societal challenges.

Furthermore, the literature review highlighted the need for a comprehensive approach to planning NbS. The six steps identified by Albert et al. (2021) focus on an adaptive planning cycle that considers the multifaceted dimensions of societal challenges. This approach necessitates interdisciplinary collaboration, bringing together planners, ecologists, and social scientists to create well-rounded NbS that considers both human and environmental aspects.

Nonetheless, the review highlighted a gap regarding the monitoring and evaluation of NbS effectiveness in addressing different societal challenges for its full implementation and operationalisation in spatial planning practice (Almassy et al. 2018; Somarakis et al. 2019; EC 2020b; Albert et al. 2021). Ecosystems' dynamic and uncertain nature requires ongoing assessment of NbS interventions to ensure their goals are being achieved. In the context of CCA, recognising the value of transformative capacity is crucial for enhancing NbS effectiveness. Transformative capacity offers a fresh perspective, enabling the development of innovative solutions to complex and persistent climate challenges (Sousa et al. 2023).

This research analysis contributed to bridge the gap in NbS implementation and operationalisation by proposing the novel three-dimensional categorisation system, since it guides in selecting the most suitable NbS principle framework for addressing specific societal challenges. While the literature review highlighted challenges in NbS implementation, selecting the appropriate framework is crucial for overcoming these hurdles. Existing frameworks offer valuable guidance but often lack a clear system for choosing the most relevant one for a particular context. By categorising the principles, the work empowers practitioners to make informed decisions and select NbS principles most suited for their specific project's ecological, social, and governance aspects, thus providing more effective NbS in spatial planning. Despite this research effort, there is still space for improvement. To further refine the framework selection process and offer a more objective tool for practitioners, future research could delve deeper through a quantitative analysis of the content within these NbS principle frameworks.

### NbS and CCA in policy

The literature review examined the integration of NbS and CCA in various global, EU, and Portuguese policy instruments, and whether NbS is considered for addressing CCA. The findings underline the importance of addressing these topics to bridge the gap between policy intentions and practical implementation. The review acknowledged the ongoing challenge of effectively monitoring and evaluating the impact of NbS before, during, and after their implementation. While NbS holds a great potential for addressing environmental and climate challenges, including the interrelated crises of climate change and biodiversity loss, the assessment of their outcomes remains a complex task. The review emphasises that a lack of coherent indicators and assessment frameworks for tracking NbS effectiveness poses a significant hurdle (Somarakis et al. 2019; Castellari et al. 2021), despite the work of the European Commission in delineating and enumerating indicators for assessing the efficacy and influence of NbS (EC 2021).

Regardless of proliferation of policies valuing NbS, the application of its principles to actual practice is often hampered by governance fragmentation and the absence of standardised evaluation methods. The study on the policies at different levels highlights the increasing recognition of NbS for CCA across various contexts. The nexus between NbS and CCA is evident in key international agreements like the UN Framework Convention on Climate Change and the Paris Agreement and the European Green Deal (Seddon et al. 2019, 2020). Despite the rhetoric, actual global and EU policies support and the extent of implementation vary substantially (Castellari et al. 2021). Inconsistent policies and fragmented governance mechanisms hinder the effective coordination and financing needed to fully leverage the potential of NbS for CCA (Trémolet 2019). According to Calliari et al. (2022), the EU policies need to be flexible enough to allow Member States to adapt policies to their local contexts, and to ensure that their objectives are achieved. Due to these challenges, integrating NbS in EU policy frameworks has resulted in a mix of mandatory or voluntary instruments within EU strategies and directives, with different levels of support.

Most EU policies, while explicitly mentioning NbS or its associated concepts, are primarily non-binding instruments related to NbS (Castellari et al. 2021; Davies et al. 2021). EU policies are widely based on voluntary actions and often lack quantitative and measurable goals for NbS development and quality (Calliari et al. 2022). For example, despite the EU Forest Strategy identifying NbS as a priority for investment, it fails to develop national adaptation strategies to integrate NbS for CCA (Calliari et al. 2022). Similarly, the EU Strategy on GI aimed to scale up ecosystem restoration and integrate NbS into various policy domains but failed to encourage action at scale (Gerritsen et al. 2021). The EU Urban Agenda, which refers to NbS, particularly GI, allows Member States to choose their priority themes and voluntarily associate with Action Plans (Calliari et al. 2022). Despite promoting NbS to address societal challenges like CCA, these policies only encourage actions rather than mandate them, relying on Member States' self-initiative and voluntary commitments (Davies et al. 2021).

Although for some authors, EU policies have been viewed as inadequate to create the necessary political will within Member States (Gerritsen et al. 2021), the EC has started introducing more enforceable and binding legal obligations regarding environmental policy frameworks to bring more nature into urban areas (Calliari et al. 2022). The EU Biodiversity Strategy for 2030 foresees developing and launching a nature restoration plan for the EU that intends to include binding targets by 2030 (EC 2020a). This plan is seen as a response to the gap in the previous strategy (EC 2011) to meet its targets due to the lack of mandatory requirements (Calliari et al. 2022). Similarly, the EU Floods Directive mandates the use of natural water retention areas, in their flood risk management plans (EC 2007), though Gerritsen et al. (2021) argue for further improvements in the EU Floods Directive regarding the use of NbS.

In addition, NbS projects require multidisciplinary approaches, especially for CCA. Hence, it is essential to connect science, policy, and practice to facilitate NbS design, implementation, and operationalisation (Somarakis et al. 2019). Nevertheless, the lack of a common language between those spheres may hinder cooperation and cause misunderstandings (Fletcher et al. 2015; Prudencio & Null 2018). Using plain language when presenting NbS projects to experts from different fields and society is crucial (Somarakis et al. 2019). As Calliari et al. (2022) stated, there is still a need for the widespread deployment and increase in the mainstreaming of NbS to address CCA across the EU policies. According to Castellari et al. (2021), other demanding gaps remain, such as the lack of EU quantitative targets (e.g., on application, coverage, and quality) and agreed standards to assess NbS progress, effectiveness and benefits, their related policies, and better communication to decision-makers.

At the national level, it was possible to understand that Portuguese policy sheds light on the need for multifaceted policy integration. The ‘National Strategy for Climate Change Adaptation’ and the ‘National Climate Law’ underscore the importance of forests and green spaces in mitigating and adapting climate impacts. The ‘Network of Municipalities for Local Adaptation to Climate Change’ emphasises the significance of localised adaptation strategies and solutions, acknowledging the critical role of NbS at the community level. The ‘National Program for Spatial Planning Policy’ further solidifies the commitment to align environmental and climate policies to foster effective NbS implementation.

Noteworthy, Portuguese water and agriculture policies still lack sufficient CCA integration, which is crucial given the importance of water, land, and food management for climate adaptation. Notably, the 2019 ‘National Program for Spatial Planning Policy’ aims to strengthen coherence between national policies and instruments, facilitating NbS implementation for CCA.

In summary, despite the growing integration of NbS and CCA in global, EU, and Portuguese policies, challenges persist in policy coherence, standardised evaluation methods, and comprehensive integration in specific sectors. The Portuguese context shows a positive trend, indicating NbS as a key CCA tool. Limited to policy analysis, this study highlights the need for on-the-ground case studies. In-depth exploration of NbS projects can reveal real-world challenges, opportunities, and real-world effectiveness of NbS implementation, informing best practices to overcome obstacles. This knowledge can guide the development of more effective policy documents, particularly at the municipal and local levels, with stronger strategies for utilising NbS to tackle CCA, ultimately enhancing community resilience.

## Conclusions

This review explored the NbS umbrella concept, its implementation, and operationalisation in spatial planning and its inclusion in different policy instruments to address CCA. To achieve the proposed objectives, a methodology comprising three distinct literature review methods, namely the NTM, SSM following PRISMA 2020 guidelines, and the SM, was applied. The NTM provided a holistic understanding of the NbS umbrella concept and identified critical research domains, while the SSM tackled limitations and uncovered knowledge gaps. The SM, on the other hand, expanded the research scope by identifying additional sources, ultimately contributing to a more comprehensive and nuanced analysis. Together, these three methods facilitated a comprehensive exploration of NbS, enabling meaningful insights and conclusions from a diverse array of sources.

The review showed that the NbS principle frameworks help better understand the NbS concept and how to implement and operationalise it and its EbAp effectively in spatial planning. Additionally, the review investigated whether they are included in policies to CCA.

The analysis of the NbS umbrella concept revealed how it is framed within different EbAps for tackling societal challenges. Despite this diversity, the study emphasises the need to prioritise trade-offs among the various NbS benefits. The concept itself also needs to be continually updated to incorporate new EbAp as they emerge.

The literature review also revealed a diversity of proposed NbS principles frameworks. Because, NbS interventions still face challenges, the study proposed three dimensions—'ecological and environmental,' 'social and cultural,' and 'urban and governance—to improve the comprehension and utility of NbS principles frameworks. Regarding the first objective, it is important to recognise the role of the IUCN Global Standard for NbS (IUCN 2020a, 2020b) and the six steps to planning NbS proposed by Albert et al. (2021) in operationalising the different NbS principles. They are instrumental in designing, implementing, and operationalising NbS interventions in spatial planning practice. The review, however, underscores the need to continually adapt and apply both NbS principles to various case studies. Overall, a thorough understanding of the NbS umbrella concept is essential for effectively applying the various principles that have been developed.

The review of policies revealed growing awareness of NbS as a tool for tackling CCA across various contexts. Nevertheless, the integration of NbS for CCA differs significantly according to the policy area and scale. For instance, the level of support and implementation varies considerably within global and EU policies. At the Portuguese level, policies point this pivotal role of NbS, but specific areas still need improvement. Despite all efforts, continued promotion of NbS and related concepts within policy documents is still needed. Additionally, efforts should focus on refining policy coordination, establishing standardised mechanisms for assessing NbS progress, and enhancing communication with key decision-makers.

This analysis of policy documents offers valuable groundwork, but further research is needed. Examining on-the-ground NbS implementation would provide invaluable insights into real-world challenges and opportunities that could better inform the development of best practices for addressing CCA and ultimately lead to the creation of more effective policies. Focusing on the municipal and local levels, these improved policies can outline stronger strategies for using NbS to tackle CCA, leading to more resilient communities.

This review significantly contributes to the growing knowledge surrounding NbS, offering insights into their implementation challenges, potential benefits, and policy integration. The comprehensive understanding gained through this study establishes a robust foundation for future endeavours, focused on unlocking the full potential of NbS to address complex societal challenges, including CCA while promoting harmonious coexistence between human activities and the environment.

Emphasising the importance of monitoring and evaluating NbS, the article suggests the need for further investigation on the effectiveness of NbS in tackling societal challenges, particularly CCA. Furthermore, analysing the diverse approaches and assessment frameworks employed to assess the effectiveness of NbS is crucial to gain valuable insights for the research community.

## Supplementary Information

Below is the link to the electronic supplementary material.

Supplementary file1 (PDF 617 kb)
