# Supplementary material for: Nature-based solutions in spatial planning and policies for climate change adaptation: A literature review
Source: Ambio. 2024 Jul 30;53(11):1599–617. doi: 10.1007/s13280-024-02052-1 (PMC11436602; doi:10.1007/s13280-024-02052-1)
Supplement: Supplementary file 1 — Supplementary file1 (PDF 617 kb) [file 13280_2024_2052_MOESM1_ESM.pdf]

Ambio

Supplementary Information

This supplementary information has not been peer-reviewed

Title: **Nature-based solutions in spatial planning and policies for climate change adaptation: a literature review**

APPENDIX S1

Table S1. Nature-based solutions concept definitions | source: (Albert et al., 2019; Balian et al., 2014; Cohen-Shacham et al., 2019; Dorst et al., 2019; EC, 2015b; Kabisch et al., 2016; Maes & Jacobs, 2015; UNEP, 2022; WWF, 2020a).

**Definitions Of Nature-Based Solutions Concept**	
**Author/ Institution/Project**	**Definition**
Balian et al. (2014)<br>BiodivERsA Project	Refers to the use of nature in tackling challenges such as climate change, food security, water resources, or disaster risk management, encompassing a broader definition of how to conserve and use biodiversity in a sustainable manner. By going beyond the threshold of traditional biodiversity conservation principles, this concept also integrates societal factors such as poverty alleviation, socio-economic development and efficient governance principles (Balian et al., 2014, p. 5).
European Commission (EC) (2015)	Solutions that are inspired and supported by nature, which are cost-effective, simultaneously provide environmental, social and economic benefits and help build resilience. Such solutions bring more and more diverse, natural features and processes into cities, landscapes and seascapes, through locally adapted, resource-efficient and systemic interventions (EC, 2015b, p. 24).
Maes and Jacobs (2015)	Defined as any transition to the use of ecosystem services with decreased input of non-renewable natural capital and increased investment in renewable natural processes (Maes & Jacobs, 2015, p. 123).
Cohen-Shacham et al. (2019)<br>International Union for Conservation of Nature (IUCN)	Defined as actions to protect, sustainably manage and restore natural or modified ecosystems, which address societal challenges (e.g., climate change, food and water security or natural disasters) effectively and adaptively while simultaneously providing human well-being and biodiversity benefits (Cohen-Shacham et al., 2019, p. 14).
Kabisch et al. (2016)<br>BIOCLIM Project	Promote the maintenance, enhancement, and restoration of biodiversity and ecosystems as a means to address multiple concerns simultaneously (Kabisch et al., 2016, p. 1).
Albert et al. (2019)	Defined as actions that (i) alleviate a well-defined societal challenge, (ii) utilize ecosystem processes of spatial, blue and green infrastructure networks, and (iii) are embedded within viable governance or business models for implementation (Albert et al., 2019, p. 14).
Dorst et al. (2019)<br>NATURVATION Project	Defined as interventions based on nature that are envisaged to address sustainability challenges such as resource shortages, flood and heat risks and ecosystem degradation caused by urbanisation and climate change (Dorst et al., 2019, p. 1).
World Wide Fund for Nature (WWF) (2020)	Defined as ecosystem conservation, management and/or restoration interventions intentionally planned to deliver measurable positive climate adaptation and/or mitigation benefits that have human development and biodiversity co-benefits managing anticipated climate risks to nature that can undermine their long-term effectiveness (WWF, 2020a, p. 3).

United Nations Environment Assembly (UNEA) (2022)	Defined as actions to protect, conserve, restore, sustainably use and manage natural or modified terrestrial, freshwater, coastal and marine ecosystems which address social, economic and environmental challenges effectively and adaptively, while simultaneously providing human well-being, ecosystem services, resilience and biodiversity benefits (UNEP, 2022, p. 2).

APPENDIX S2

Table S2. The ecosystem-based approaches from the nature-based solutions | source: Agarwal et al. (2000); Barot et al. (2012); Benedict and McMahon (2006); Castellari et al. (2021); CBD (2004, 2009, 2016); Cicin-Sain and Knecht (1998); Costanza et al. (1997); Dorst et al. (2019); EC (2013, 2015a); EPA (2000); Fletcher et al. (2015); Griscom et al. (2017); Griscom et al. (2020); Helsinki (1993); Hewett et al. (2020); IUCN WCPA (2019); Lavorel et al. (2015); Mansourian and Parrotta (2008); Sudmeier-Rieux et al. (2019); UNEP-WCMC (2019); UNEP/CBD (2000); Wilkie et al. (2003); Wong (2006). Categories according to Cohen-Shacham et al. (2019); Cohen-Shacham et al. (2016); EC (2021).

**NATURE-BASED SOLUTIONS ECOSYSTEM-BASED APPROACHES**		
**CATEGORY**	**APPROACH**	**DEFINITION**
Ecosystem protection and restoration approaches	Ecological Restoration (ER)	Ecological restoration refers to the process of managing or assisting the recovery of an ecosystem that has been degraded, damaged, or destroyed as a means of sustaining ecosystem resilience and conserving biodiversity. Degradation is characterized by a decline or loss of biodiversity or ecosystem functions. Degradation and restoration are context-specific and refer to both ecosystems' state and ecosystem processes (CBD, 2016, p. 4).
	Ecological Engineering (EE)	Aims to develop a more sustainable practice informed by ecological knowledge regarding the protection and restoration of ecological systems, their modification to increase the quantity, quality and sustainability of particular services they provide or building new ecological systems that provide services that would otherwise be provided through conventional engineering based on non-renewable resources (Barot et al., 2012).
	Catchment Systems Engineering (CSE)	CSE is a proactive interventionist approach to modifying runoff at the catchment scale instigated and delivered by stakeholders. It provides a holistic way of addressing multiple issues such as hydrological extremes, diffuse pollution, and soil erosion simultaneously by combining multiple small-scale interventions distributed throughout catchments with larger-scale traditional engineering structures. Small-scale features typically mimic natural processes, primarily targeting hydrological flow pathways such as overland flow, field drain, and ditch function, while more extensive features such as wetlands, retention basins, and levees typically provide additional protection for assets and urban areas (Hewett et al., 2020, p. 2) .
	Forest Landscape Restoration (FLR)	Forest landscape restoration is a planned process that aims to regain ecological integrity and enhance human wellbeing in deforested or degraded landscapes (Mansourian & Parrotta, 2008, p. 2). FLR is the ongoing process of recovering ecological functionality and improving human well-being across deforested

		or degraded forest landscapes. FLR is more than just planting trees – it is restoring a whole landscape to meet present and future needs and offer multiple benefits and land uses over time[1].
	Area-based Conservation (AbC)	AbC is a term under the Convention on Biological Diversity (CBD) that refers particularly to protected areas (PAs), other effective area-based conservation measures (OECMs), area-based conservation management or governance approaches, and Areas of importance for biodiversity and ecosystem services (IUCN WCPA, 2019; UNEP-WCMC, 2019).
Issue-specific ecosystem-related approaches	Ecosystem Approach (EA)	The ecosystem approach is a strategy for the integrated management of land, water and living resources that promotes conservation and sustainable use in an equitable way. An ecosystem approach is based on applying appropriate scientific methodologies focused on levels of biological organization, which encompass the essential structure, processes, functions and interactions among organisms and their environment. It recognizes that humans, with their cultural diversity, are an integral component of many ecosystems (UNEP/CBD, 2000, pp. 103-104).
	Ecosystem-based Adaptation (EbA)	Integrates the sustainable use of biodiversity and ecosystem services into an overall adaptation strategy to help people to adapt to the adverse effects of climate change that can be cost-effective and generate social, economic and cultural co-benefits and contribute to the conservation of biodiversity (CBD, 2009; Dorst et al., 2019).
	Ecosystem-based Mitigation (EbM)	The use of ecosystems for their carbon storage and sequestration service to aid climate change mitigation (Sudmeier-Rieux et al., 2019).
	Ecosystem-based Disaster Risk Reduction (Eco-DRR)	It entails combining natural resources management approaches, or the sustainable management of ecosystems, with disaster risk reduction (DRR) methods, such as early warning systems and emergency planning, to have more effective disaster prevention, reduce the impact of disasters on people and communities, and support disaster recovery (Sudmeier-Rieux et al., 2019).
	Ecosystem Services (ES) (approach/framework)	Defined as the ecosystem goods (such as food) and services (such as waste assimilation) and represent the benefits human populations derive, directly or indirectly, from ecosystem functions (Costanza et al., 1997, p. 253).
	Natural Water Retention	NWRM are multifunctional measures that aim to protect water resources and address water-related challenges by restoring or

[1] https://infoflr.org/index.php/what-flr

	Measures (NWRM)	maintaining ecosystems as well as natural features and characteristics of water bodies using natural means and processes. It enhances the retention capacity of aquifers, soil, and aquatic and water-dependent ecosystems with a view to improving their status. This approach can supports, for example, green infrastructure by improving the qualitative and quantitative status of water bodies and reducing vulnerability to droughts and floods (EC, 2015a).
	Climate Adaptation Services (CAS)	Defined as the benefits to people from the increased social ability to respond to change, provided by the capacity of ecosystems to moderate and adapt to climate change and variability (Lavorel et al., 2015, p. 13) . CAS complements the EbA approach and helps people develop solutions for adaptation to climate change.
	Sustainable Climate Action (SCA)	SCA is defined as a transition employing nature-based solutions, alongside a rapid phase-out of fossil fuel use, to reduce the scale and impacts of climate change, while providing positive benefits for biodiversity and other sustainable development goals (CBD, 2004).
	Natural Climate Solutions (NCS)	NCS are defined as conservation, restoration, and improved land management actions that increase carbon storage and/or avoid greenhouse gas emissions across global forests, wetlands, grasslands, and agricultural lands (Griscom et al., 2017) . Recently it was clarified that nature climate solutions can also be referred to as nature-based solutions (Griscom et al., 2020).
Infrastructure-related approaches	Natural Infrastructure (NI)	Natural infrastructure is defined as a strategically planned and managed network of natural lands, such as forests and wetlands, working landscapes, and other open spaces, that conserves or enhances ecosystem values and functions and provides associated benefits to human populations (Benedict & McMahon, 2006).
	Green-Blue Infrastructure (GBI)	Defined as a strategically planned network of natural and semi-natural areas with other environmental features designed and managed to deliver a wide range of ecosystem services. It incorporates green spaces (or blue if aquatic ecosystems are concerned) and other physical features in terrestrial (including coastal) and marine areas. On land, green infrastructure is present in rural and urban settings (EC, 2013).
Ecosystem-based management approaches	Integrated Coastal Management (ICM)	ICM is a process by which rational decisions are made regarding the conservation and sustainable use of coastal and ocean resources and their space. The process aims to overcome the fragmentation inherent in single-sector management approaches (e.g., fishing operations, oil and gas development), in the divisions in jurisdiction among different levels of government and the land-water interface (Cicin-Sain & Knecht, 1998).

Integrated Water Resources Management (IWRM)	IWRM is a process that promotes the co-ordinated development and management of water, land, and related resources to maximize the resultant economic and social welfare in an equitable manner without compromising the sustainability of vital ecosystems (Agarwal et al., 2000, p. 22).
Sustainable Urban Drainage Systems (SUDS)	SUDS is designed with a range of technologies and practices to attenuate stormwater runoff, reduce piping network pressure, and mitigate the environmental impact of non-point source pollution (Fletcher et al., 2015).
Low Impact Development (LID)	LID refers to systems and practices that use or mimic natural processes that result in infiltration, evapotranspiration, or stormwater to protect water quality and associated aquatic habitat (EPA, 2000).
Water Sensitive Urban Design (WSUD)	Defined as the integration of urban planning with the management, protection and conservation of the urban water cycle, ensuring that urban water management is sensitive to natural hydrological and ecological processes (Wong, 2006).
Best Management Practices (BMPs)	It is a concept used to describe a type of water pollution control. BMPs describe ways to manage the land and activities to mitigate pollution of surface and groundwater (Fletcher et al., 2015).
Sustainable Management (SM)	Sustainable management refers to the sustainable management of ecosystems, water, forests, and natural resources. By making use of SM can build resilience in ecosystems. It is based on the twelve guiding principles for achieving sustainable management (CBD, 2004). SM involve integrated, holistic approaches to management that consider the interdependence of human activities, ecosystems and human well-being, taking a long-term outlook across different spatial scales (Castellari et al., 2021).
Sustainable Forest Management (SFM)	The term SFM is under the umbrella of sustainable management (SM), and it was developed according to the Rio forest principles, which aimed to apply the ecosystem approach for forests (Wilkie et al., 2003). SFM can be defined as a stewardship and use of forest lands in a way and at a rate that maintains their biodiversity, productivity, regeneration capacity, vitality and their potential to fulfil, now and in the future, relevant ecological, economic and social functions, at local, national and global levels and that does not cause damage to other ecosystems (Helsinki, 1993).

APPENDIX S3

Table S3. Nature-base solutions principles from different frameworks before and after the establishment of the IUCN principles framework in 2016 by the Resolution WCC-2016-Res-069 | Source: Albert et al. (2021); CBD (2004); Cohen-Shacham et al. (2019); Dorst et al. (2019); Dumitru et al. (2020); Farrugia and Gallina (2008); Hansen et al. (2017); (IUCN, 2012); IUCN (2016); Kabisch et al. (2022); Woodhouse et al. (2015); WWF (2020b).

**Nature-Based Solutions Principles frameworks**			
**Year**	**Author/Institutions**	**Objective, scope and dimension**	**Principles**
**Before IUCN principles**			
2004	**Convention on Biological Diversity (CBD) guidelines** – Ecosystem Approach	**Objective:** It is focused on guiding the sustainable management and conservation of ecosystems and their biodiversity. These principles provide a comprehensive framework for how ecosystems should be managed and highlight key considerations and approaches.<br>**Scope:** The scope of these principles is broad and covers various aspects of ecosystem management, conservation, and sustainability, with an emphasis on inclusivity, adaptability, and the long-term well-being of both ecosystems and human societies.	**The twelve principles of the ecosystem approach for ecosystem management (CBD, 2004) :**<br>1. Highlights the importance of indigenous population for the management of the ecosystem, that should be done according to ecosystems intrinsic values and benefits for humans, fairly and equitably.<br>2. Recognises the involvement of all stakeholders in decentralised systems for more efficiency, effectiveness, and equity of ecosystem use.<br>3. Ecosystem's managers should acknowledge the effects of their activities on territory and adjacent ecosystems:<br>4. Recognises that any ecosystem management programme should reduce market distortions that affect biological diversity, regulate incentives to improve biodiversity conservation and sustainable use, and internalize ecosystems costs and benefits.<br>5. Prioritise the conservation of ecosystems structure and function to maintain ecosystem services provision.<br>6. Ecosystems management should be cautious due to the limits of ecosystems productivity capacity.<br>7. The ecosystem management should be attempted at the appropriate spatial and temporal scale, ensuring the connection between ecosystems for their gene exchange.<br>8. Recognises the temporal scale and lag-effects of ecosystem processes, so the management needs to be set for the long term.

			9. Management should be adaptive to anticipate ecosystem changes and consider mitigation actions to cope with climate change.<br>10. There is a need for a shift from management based on protection or non-protection of biodiversity to an integrative management balance on conservation and use of biological diversity.<br>11. Recognises the importance to consider all forms of information (e.g., scientific, indigenous, and local knowledge), innovations and practices.<br>12. Recognises the importance of involving the expertise and stakeholders at the local, national, regional, and international level to solve biological diversity management problems.
2012	**The IUCN Programme 2013–2016**	**Objective:** It is to offer guidance on what types of interventions should be considered as NbS and what characteristics they should possess. These principles are intended to shape the approach and characteristics of NbS in addressing global challenges while harnessing the potential of natural ecosystems.<br>**Scope:** The scope of these principles is to guide the development and implementation of NbS projects that effectively harness the benefits of nature to address global challenges while considering economic, social, and environmental factors.	**The seven principles that provided guidance on what type of interventions could (or should not) be considered as an NbS (IUCN, 2012):**<br>1. NbS delivers effective solutions to global challenges through nature.<br>2. NbS produce various benefits through ecosystem services provided by their resilient and well-functioning ecosystems.<br>3. NbS are cost-effective compared to other solutions that involve costly and vulnerable infrastructure investments, unlike NbS, which often builds resilience at less cost.<br>4. NbS interventions must be easily and compellingly communicated to the audiences.<br>5. NbS need to be measured, verified, and replicated.<br>6. The implementation of NbS needs to be managed concerning the scope and extent of communities' dependency on natural resources, respecting their gender, ethnicity, and social groups.<br>7. NbS must be attractive to public and private investors, producers, and consumers (e.g., through payments for ecosystem services and other incentive mechanisms).
2015	**Imperial College London** - Guiding principles for evaluating the impacts of	**Objective:** It is to provide a comprehensive framework for guiding conservation efforts that aim to improve the well-being of local communities. These principles emphasize	**The nine key principles used by conservation interventions to enhance human well-being (Woodhouse et al., 2015):**

	conservation interventions on human well-being	the importance of taking a holistic and community-centred approach to conservation.<br><br>**Scope:** The scope of these principles is to guide conservation interventions in a way that not only benefits the environment but also enhances the well-being of local communities, taking into account the complexity and dynamics of human well-being and the importance of community involvement and ethical research practices.	1. Local people are at the centre of the evaluation since they are the most affected by policy changes and interventions.<br>2. Given the multi-dimensional nature of human well-being, it is essential to measure all the outcomes through different indicators.<br>3. The process of attributing specific effects, i.e., changes in human well-being, should be at the core of the evaluation process.<br>4. It is important to understand change processes that provide evidence of causal linkages that lead to human well-being.<br>5. Consider trajectories of change since well-being is an ongoing dynamic process.<br>6. Develop investigation regarding institutions and governance structures since human well-being depends on institutions that promote relationships between individuals and groups and humans and ecosystems.<br>7. Select and apply methods and toolkits that conservation evaluators can draw upon in collecting data on well-being indicators and contextual and confounding factors.<br>8. Consider heterogeneity within the target group since there are trade-offs between the interventions that provide human well-being and people and between or within communities.<br>9. Ensure that the quality of research is defined by how the research is conducted and the relationships between the researchers and participants.
**IUCN preliminary principles establishment**			
2016	**Resolution "WCC-2016-Res-069-EN** - Defining Nature-based Solutions"	**Objective:** It is to provide a framework and guidance for understanding what NbS entail and how they should be implemented. These principles emphasize the importance of integrating nature conservation into various aspects of societal development and sustainability.	**The eight preliminary principles considered in conjunction with NbS definition (IUCN, 2016):**<br>1. NbS embrace nature conservation norms (and principles).<br>2. NbS can be implemented alone or in an integrated manner with other solutions to societal challenges (e.g., technological and engineering solutions).

		**Scope:** The scope of these principles is to guide the development and implementation of NbS as a holistic and integrated approach to addressing societal challenges while considering ecological, cultural, and social dimensions. These principles emphasize the importance of context-specific, equitable, and sustainable solutions that embrace and work in harmony with nature.	3. NbS are determined by site-specific natural and cultural contexts that include traditional, local and scientific knowledge.<br>4. NbS produce societal benefits in a fair and equitable way in a manner that promotes transparency and broad participation.<br>5. NbS maintain biological and cultural diversity and the ability of ecosystems to evolve over time.<br>6. NbS are applied at a landscape scale.<br>7. NbS recognise and address the trade-offs between the production of a few immediate economic benefits for development, and future options for the production of the full range of ecosystems services.<br>8. NbS are an integral part of the overall design of policies, and measures or actions, to address a specific challenge.
**After IUCN principles**			
2017	**GREEN SURGE Project** - Urban Green Infrastructure Planning: A Guide for Practitioners	**Objective:** It is to provide a framework for the effective planning and implementation of green infrastructure in urban areas. These principles emphasize the integration of natural elements into urban planning to enhance sustainability and improve the quality of life for urban residents.<br>**Scope:** The scope of these principles is to guide urban planners, policymakers, and stakeholders in developing UGI that is integrated, connected, multifunctional, socially inclusive, and considers various scales and types of green spaces. These principles aim to create sustainable and resilient urban environments that benefit both nature and urban communities.	**The four interlinked principles that underpin the best practices to Urban Green Infrastructure planning (Farrugia & Gallina, 2008; Hansen et al., 2017):**<br>1. Green-grey integration - integration and coordination of green infrastructure (e.g., urban green spaces) with grey infrastructure (e.g., transport systems and utilities).<br>2. Connectivity – to create and restore connections between green spaces to support and protect their processes, functions and benefits provided.<br>3. Multifunctionality – combine different functions to strengthen the capacity of urban green spaces in delivering ecosystem services, creating synergies, and reducing conflicts and trade-offs.<br>4. Social inclusion – the planning processes are open and incorporate the knowledge and needs of different parties.<br>**Supporting principles should also be considered for UGI planning, such as (Hansen et al., 2017):**<br>1. Multi-scale – to link different spatial levels (e.g., metropolitan regions and individual sites).

			2. Multi-object: UGI planning should consider all types of urban green and blue spaces as part of a green infrastructure network regardless of ownership and/origin.<br>3. Inter and transdisciplinary – to link different disciplines (e.g., ecology, urban and regional planning, landscape architecture) science, policy, practice and ideally developed in partnership between local authorities and other stakeholders.
2019	**IUCN**	**Objective:** It is to provide a comprehensive framework for guiding the adoption and expansion of NbS as a sustainable approach to addressing global challenges. These principles emphasize the integration of nature-based approaches into various aspects of policy and planning for more effective and equitable solutions.<br>**Scope:** The scope of these principles is to guide decision-makers, policymakers, and stakeholders in adopting and scaling up NbS as a holistic and integrated approach to addressing global challenges while considering ecological, social, and economic dimensions. These principles aim to create sustainable and resilient solutions that benefit both nature and human well-being while upholding conservation and equity principles.	**The eight core principles developed for successfully implementing and upscaling NbS (Cohen-Shacham et al., 2019):**<br>1. Nature-based solutions embrace nature conservation norms and principles and can be complementary to them and benefit from them.<br>2. Nature-based solutions can be implemented alone or integrated beside other solutions to tackle global societal challenges and providing a full range of ecosystem services due to their umbrella framework that includes a mixture of different concepts.<br>3. Nature-based solutions are settled by the natural and cultural contexts that include traditional, local, and scientific knowledge through people's lives and ecosystem use.<br>4. Nature-based solutions promote fair and equitable societal benefits (e.g., provisioning and/or cultural ecosystem services, such as food and/or recreational spaces) in a way that improves transparency and broad participation.<br>5. Nature-based solutions provide biological and cultural diversity and ecosystems' ability to develop over time to maintain biological and cultural diversity and be resilient for future environmental change.<br>6. Nature-based solutions should be applied at the landscape scale, regardless of the implementation scale (e.g., large spatial scales that combine different ecosystems or specific local levels). It is necessary to consider their wider landscape-scale context and consequences, aiming to upscale up where appropriate.<br>7. Nature-based solutions consider and address the trade-offs between the immediate production of economic benefits and the future total production of ecosystem services. Therefore, NbS should not change an

			existed ecosystem to favour the provision of a particular service (e.g., replacing natural mixed woodland with a monoculture tree plantation). When deciding among the different NbS, it is essential to understand the trade-offs between the present and future benefits to maintain or enhance the ecosystem services.<br>8. Nature-based solutions should be incorporated in policies design, measures, and actions to address a specific challenge.
	**NATURVATION Project** - Urban greening through nature-based solutions: key characteristics of an emerging concept	**Objective:** It is to provide a foundational framework for understanding and applying NbS effectively. These principles emphasize the key characteristics and considerations that underlie the concept of NbS.<br>**Scope:** The scope of these principles is to guide the planning and implementation of NbS by emphasizing the fundamental role of nature, the multifaceted nature of NbS solutions, the need for integrated governance, and the importance of tailoring NbS to the specific context in which it is applied. These principles are aimed at fostering sustainability, resilience, and effective responses to societal challenges through the utilization of natural elements and processes.	**The four core principles of NbS planning and implementation (Dorst et al., 2019):**<br>1. Nature as the concept' s central foundation that can take many forms.<br>2. NbS is characterised by its multifunctionality and solution orientation since it simultaneously addresses several social, economic, and environmental challenges.<br>3. NbS requires implementation through holistic and integrative governance and planning approaches since it integrates multiple values and disciplines.<br>4. NbS considers the importance of adaptation to place-based conditions since it regards its dependence and effects on the socio-spatial environment.
2020	**Connecting Nature project** - Identifying principles for the design of robust impact evaluation frameworks for nature-based solutions in cities	**Objective:** It is to provide guidance for the conceptualization and design of evaluation schemes specifically for Nature-based Solutions (NbS) in urban areas, particularly in cities. These principles are intended to inform the process of assessing and monitoring the outcomes and impacts of NbS initiatives in urban environments.	**The four principles to guide the conceptualization and design of evaluation schemes of NbS in cities (Dumitru et al., 2020):**<br>1. Conceptualize and test hypotheses through which NbS deliver outcomes.<br>2. Evaluate the potential interactions between the types of impacts and identify the social and environmental factors that lead to them.

		**Scope:** The scope of these principles is to guide the process of evaluating NbS initiatives in urban settings, with a particular focus on cities. They aim to ensure that evaluations are systematic, comprehensive, and inclusive, taking into account the complexity of urban systems and the diverse perspectives of various stakeholders. The principles also highlight the importance of considering both short-term and long-term impacts when assessing the effectiveness of NbS interventions.	3. The evaluation process should include methodologies that enable different social groups to engage with NbS to assess the actual magnitude of the impact from their outcomes.<br>4. The evaluation should include the impacts assessments done over time for each outcome to build a solid evidence-based between the different cases implemented across Europe.
	**WWF** - nature-based solutions for climate change	**Objective:** It is to provide a comprehensive framework for the planning and implementation of NbS initiatives to address climate change challenges. These principles emphasize the importance of science-based, collaborative, and measurable approaches to climate change mitigation and adaptation.<br>**Scope:** The scope of these principles is to guide the development and implementation of NbS as a holistic and science-based approach to climate change mitigation and adaptation. These principles underscore the importance of collaboration, accountability, and evidence-based decision-making in addressing the complex challenges of climate change while benefiting both nature and society.	**The five key principles for nature-based solutions for climate change (WWF, 2020b):**<br>1. NbS increases climate change adaptation and/or mitigation and ecosystems functionality.<br>2. NbS uses the best available science (e.g., climate, biological and social) to set achievable and measurable targets.<br>3. NbS instigates synergies since it helps reduce and/or avoid emissions, reduce human vulnerability to risk, and conserve nature and ecosystem services trade-offs.<br>4. NbS promotes a partnership between Indigenous people and local stakeholders to co-design and co-implement solutions based on their co-responsibility for societal challenges resolution.<br>5. NbS outcomes should be measurable and traceable through robust monitoring, evaluation and reporting frameworks.
2021	**Global Land Project (GLP)** - Planning nature-based solutions:	**Objective:** It is to provide a comprehensive framework for effective NbS planning and execution. These principles are aimed at ensuring that NbS initiatives are	**The five key guiding principles that may enhance the successful NbS implementation (Albert et al., 2021):**

	Principles, steps, and insights	contextually appropriate, evidence-based, integrated, equitable, and involve transdisciplinary collaboration.<br><br>**Scope:** The scope of these principles is to guide the development and implementation of NbS initiatives that are contextually relevant, evidence-based, comprehensive, equitable, and foster collaboration among various stakeholders. These principles aim to promote the successful integration of NbS into broader sustainability strategies while addressing the specific needs and challenges of each location.	1. Place-specificity since both societal challenges and potential NbS are bond to a specific place and context.<br>2. Evidence base - it is important to have available information and knowledge to infer reliable recommendations and actions for NbS planning.<br>3. Integration - to consider related approaches (e.g., ES, GBI, EE and/or natural capital), temporal, spatial and sectoral scales within NbS planning and policies in the governance context.<br>4. Equity - to recognize the rights, values and interests of the different stakeholders involved in the NbS implementation and its pre-existed political, economic, and social context.<br>5. Transdisciplinary - cooperation between researchers from different disciplines and non-academic stakeholders aiming to create new knowledge and contribute to NbS planning and implementation (Tress, Tress, and Fry (2005) in Albert et al. (2021)).
2022	**Principles for urban nature-based solutions**	**Objective:** It is to provide a guiding framework for the effective integration of NbS in urban environments. These principles aim to foster a systemic, inclusive, and sustainable approach to urban planning and governance.<br><br>**Scope:** The scope of these principles is to guide urban planners, policymakers, and stakeholders in integrating NbS effectively into urban planning and governance processes. By adopting a systemic, inclusive, and context-sensitive approach, urban areas can harness the benefits of NbS for both human well-being and biodiversity conservation while fostering sustainability and resilience in cities. Additionally, the emphasis on communication and learning encourages ongoing adaptation and	**The five key principles for urban NbS planning and governance (Kabisch et al., 2022):**<br>1. Need for a systemic understanding: urban NbS are integrated solutions and need to be based on a systems approach.<br>2. Benefiting people and biodiversity: urban NbS need to ensure a balanced delivery of multiple benefits for humans and non-humans.<br>3. Inclusive solutions for the long-term: urban NbS need to be inclusively designed, planned, implemented, and managed to appreciate long-term benefits.<br>4. Context consideration: urban NbS should respect and planned considering the local context.<br>5. Communication and learning: urban NbS should support mutual learning for sustainability transitions in cities.

	improvement in NbS planning and implementation.	

APPENDIX S4

Table S4. Global, European Union and Portuguese policy frameworks regarding the use of nature-based solutions (NbS) to climate change adaptation (CCA). Legend: "x" means that the policy explicit use or refer nature-based solutions (NbS) and/or climate change adaptation (CCA). "N/A" means that the policy don' t use or refer nature-based solutions (NbS) and/or climate change adaptation (CCA). The "year" in parenthesis regards the last review made to the policy. The "?" means the non-existent information regarding the policy use of nature-based solutions (NbS) and/or climate change adaptation (CCA) | sources: All the global, European Union and Portuguese policies consulted; Castellari et al. (2021) and Davis et al. (2018).

Policy Area	Global Policy	NbS	CCA	Eu Policy	NbS	CCA	National Policy	NbS	CCA
Biodiversity and forestry	a) Ramsar Convention (1975)<br>b) United Nations Convention on Biological Diversity (1993)	a) x<br>b) x	a) x<br>b) x	a) Habitats Directive (1992)<br>b) Birds Directive (2009)<br>c) EU Forest Strategy (2013)<br>d) EU Strategy on Green Infrastructure (2013)<br>e) Land Use, Land-Use Change and Forestry (LULUCF) Regulation (2018)<br>f) Biodiversity strategy for 2030 (2020)	a) N/A<br>b) N/A<br>c) x<br>d) x<br>e) x<br>f) x	a) N/A<br>b) N/A<br>c) x<br>d) x<br>e) x<br>f) x	a) Environmental Policy Law (2014)<br>b) Forest National Strategy (2015)<br>c) Nature Conservation and Biodiversity National Strategy for 2030 (2018)	a) x<br>b) x<br>c) x	a) x<br>b) x<br>c) x
Water and agriculture	N/A	N/A	N/A	a) Nitrates Directive (1991)<br>b) Water Framework Directive (2000)<br>c) Floods Directive (2007)	a) N/A<br>b) x<br>c) x<br>d) x<br>e) x	a) N/A<br>b) x<br>c) x<br>d) x<br>e) x	a) Common Agricultural Policy for Portugal (2023)<br>b) Legal Regime for the National	a) ?<br>b) x<br>c) x	d) x<br>e) N/A<br>f) N/A

				d) Common Agricultural Policy (2023)<br>e) Farm-to-fork strategy (2020)			Agricultural Reserve (2015)<br>c) Water Law (2017)		
Maritime	a) United Nations Convention on the Law of the Sea (UNCLOS) (1982)	N/A	N/A	a) Marine Strategy Framework Directive (MSFD) (2017)	a) x	a) N/A	a) National Maritime Space Planning and Management Law (2017)<br>b) Marine Environment Law (2017)	a) x<br>b) x	a) x<br>b) N/A
Climate	a) United Nations Framework Convention on Climate Change (1994)<br>b) Paris Agreement (2015)<br>c) Sendai Framework for Disaster Risk Reduction 2015-2030 (2015)	a) x<br>b) x<br>c) x	a) x<br>b) x<br>c) x	a) Action plan on the Sendai Framework for Disaster Risk Reduction (2016)<br>b) EU Strategy on Adaptation to Climate Change (2021)	a) x<br>b) x	a) x<br>b) x	a) National Strategy for Climate Change Adaptation for 2025 (2020)<br>b) Climate Law (2021)	a) x<br>b) x	a) x<br>b) x
Urban	a) New Urban Agenda – Habitat III (2016)	a) x	a) x	a) Urban Agenda for the EU (i.e., Pact of Amsterdam, 2016)<br>b) Leipzig Charter (2020)<br>c) Territorial Agenda (2030)	a) x	a) x	a) Sustainable Cities Strategy for 2020 (2015)<br>b) Law for Public Policy on Soils, Land Use and Urbanism (2017)<br>c) National Program for Territorial Cohesion (2016)<br>d) National Program for Spatial	a) x<br>b) x<br>c) x<br>d) x	a) x<br>b) x<br>c) N/A<br>d) x

							Planning Policy (2019)		
Cross-cutting	a) United Nations Convention to Combat Desertification (1996)<br>b) 2030 Agenda for Sustainable Development, Sustainable Development Goals (2015)	a) x<br>b) N/A	a) x<br>b) x	a) EU Bioeconomy Strategy (2018)<br>b) European Green Deal (2019)	a) x<br>b) x	a) x<br>b) x	a) Legal Regime for the National Ecological Reserve (2019)<br>b) National Strategy for a Sustainable Bioeconomy 2030 (in development)	a) x<br>b) ?	a) x<br>b) ?

APPENDIX S5

Table S5. List of abbreviations

ABBREVIATION	MEANING
CC	Climate Change
CCA	Climate Change Adaptation
CCAM	Climate Change Adaptation and Mitigation
EbAp	Ecosystem-based Approaches
EC	European Commission
ES	Ecosystem Services
EU	European Union
GI	Green Infrastructure
GLP	Global and Land Project
GS	Global Standard
IEEP	Institute for European Environmental Policy
IPBES	Intergovernmental Science-Policy Platform on Biodiversity and Ecosystem Services
IPCC	Intergovernmental Panel on Climate Change
IUCN	International Union for Conservation of Nature
KBA	Key Biodiversity Areas
MES	Municipal Ecological Structures
NbS	Nature-based Solutions
NPSPP	National Program for Spatial Planning Policy
NTM	Narrative or Traditional Method
PRISMA	Preferred Reporting Items for Systematic Reviews and Meta-Analyses
SCA	Sustainable Climate Action
SLM	Snowballing literature Method
SSM	Semi-systematic Method
UNDP	United Nations Development Programme
UNEA	United Nations Environment Assembly
UNEP	United Nations Environment Programme
WB	World Bank
WoS	Web of Science

WWF	World Wildlife Fund

References

Agarwal, A., Angeles, M. S. d., Bhatia, R., Chéret, I., Davila-Poblete, S., Falkenmark, M., Villarreal, F. G., Jønch-Clausen, T., Kadi, M. A., Kindler, J., Rees, J., Roberts, P., Rogers, P., Solanes, M., & Wright, A. (2000). Integrated Water Resources Management. In (pp. 71). Global Water Partnership - Technical Advisory Committee (TAC). https://hdl.handle.net/10535/4986

Albert, C., Brillinger, M., Guerrero, P., Gottwald, S., Henze, J., Schmidt, S., Ott, E., & Schröter, B. (2021). Planning nature-based solutions: Principles, steps, and insights. *Ambio*, *50*(8), 1446-1461. https://dx.doi.org/10.1007/s13280-020-01365-1

Albert, C., Schröter, B., Haase, D., Brillinger, M., Henze, J., Herrmann, S., Gottwald, S., Guerrero, P., Nicolas, C., & Matzdorf, B. (2019). Addressing societal challenges through nature-based solutions: How can landscape planning and governance research contribute? *Landscape and Urban Planning*, *182*, 12-21. https://dx.doi.org/10.1016/j.landurbplan.2018.10.003

Balian, E., Eggermont, H., & Le Roux, X. (2014). Outputs of the strategic foresight workshop ‘‘nature-based solutions in a BiodivERsA context’’. *Brussels: BiodivERsA Workshop Report*, 45. https://www.biodiversa.org/687/download

Barot, S., Lata, J.-C., & Lacroix, G. (2012). Meeting the relational challenge of ecological engineering within ecological sciences. *Ecological Engineering*, *45*, 13-23. https://doi.org/10.1016/j.ecoleng.2011.04.006

Benedict, M. A., & McMahon, E. T. (2006). *Green Infrastructure: Linking Landscapes and Communities*. Island Press. https://islandpress.org/books/green-infrastructure

Castellari, S., Zandersen, M., Davis, M., Veerkamp, C., Förster, J., Marttunen, M., Mysiak, J., Vandewalle, M., & Picatoste, J. R. (2021). *Nature-based solutions in Europe : Policy, knowledge and practice for climate change adaptation and disaster risk reduction* (EEA Report No 1/2021., Issue. https://data.europa.eu/doi/10.2800/919315

CBD. (2004). CBD Guidelines – The Ecosystem Approach. In (pp. 53). Montreal: Convention on Biological Diversity (CBD). https://www.cbd.int/doc/publications/ea-text-en.pdf.

CBD. (2009). Connecting Biodiversity and Climate Change Mitigation and Adaptation: Report of the Second Ad Hoc Technical Expert Group on Biodiversity and Climate Change. In S. o. t. C. o. B. Diversity (Ed.), *Technical Series No. 41* (pp. 126). Convention on Biological Diversity. https://portals.iucn.org/library/node/28774

CBD. (2016). Decision adopted by the Conference of the Parties to the Convention on Biological Diversity xiii/5. Ecosystem restoration: short-term action plan. In (pp. 10). Convention on Biological Diversity (CBD). https://www.cbd.int/decisions/cop/13/5

Cicin-Sain, B., & Knecht, R. (1998). *Integrated Coastal and Ocean Management: Concepts And Practices*. Island Press. https://islandpress.org/books/integrated-coastal-and-ocean-management

Cohen-Shacham, E., Andrade, A., Dalton, J., Dudley, N., Jones, M., Kumar, C., Maginnis, S., Maynard, S., Nelson, C. R., Renaud, F. G., Welling, R., & Walters, G. (2019). Core principles for successfully implementing and upscaling Nature-based Solutions. *Environmental Science & Policy*, *98*, 20-29. https://doi.org/10.1016/j.envsci.2019.04.014

Cohen-Shacham, E., Walters, G., Maginnis, S., & Janzen, C. (2016). *Nature-based Solutions to address global societal challenges*. International Union for Conservation of Nature (IUCN). https://doi.org/10.2305/IUCN.CH.2016.13.en

Costanza, R., d'Arge, R., de Groot, R., Farber, S., Grasso, M., Hannon, B., Limburg, K., Naeem, S., O'Neill, R. V., Paruelo, J., Raskin, R. G., Sutton, P., & van den Belt, M. (1997). The value of the world's ecosystem services and natural capital. *Nature*, *387*(6630), 253-260. https://doi.org/10.1038/387253a0

Davis, M., Abhold, K., Mederake, L., & Knoblauch, D. (2018). *Nature-based Solutions in European and National Policy Frameworks*. European Commission. https://networknature.eu/sites/default/files/uploads/nature-based-solutions-european-and-national-policy-frameworks.pdf

Dorst, H., van der Jagt, A., Raven, R., & Runhaar, H. (2019). Urban greening through nature-based solutions – Key characteristics of an emerging concept. *Sustainable Cities and Society*, *49*, 101620. https://doi.org/https://doi.org/10.1016/j.scs.2019.101620

Dumitru, A., Frantzeskaki, N., & Collier, M. (2020). Identifying principles for the design of robust impact evaluation frameworks for nature-based solutions in cities. *Environmental Science & Policy*, *112*, 107-116. https://doi.org/10.1016/j.envsci.2020.05.024

EC. (2013). *Green infrastructure (GI) — Enhancing Europe's natural Capital*. European Commision Retrieved from https://eur-lex.europa.eu/legal-content/EN/TXT/?uri=CELEX:52013DC0249

EC. (2015a). *EU policy document on natural water retention measures : by the drafting team of the WFD CIS Working Group Programme of Measures (WG PoM)*. Publications Office. https://data.europa.eu/doi/10.2779/396202

EC. (2015b). *Towards an EU research and innovation policy agenda for nature-based solutions & re-naturing cities. Final report of the Horizon 2020 expert group on nature-based solutions and re-naturing cities*. Publications Office. https://data.europa.eu/doi/10.2777/479582

EC. (2021). *Evaluating the Impact of Nature-based Solutions: A Handbook for Practitioners*. Publications Office of the European Union. https://data.europa.eu/doi/10.2777/244577

EPA. (2000). *Low Impact Development (LID) A Literature Review*. O. o. W. United States Environmental Protection Agency. https://nepis.epa.gov/Exe/ZyNET.exe/P1001B6V.TXT?ZyActionD=ZyDocument&Client=EPA&Index=2000+Thru+2005&Docs=&Query=&Time=&EndTime=&SearchMethod=1&TocRestrict=n&Toc=&TocEntry=&QField=&QFieldYear=&QFieldMonth=&QFieldDay=&IntQFieldOp=0&ExtQFieldOp=0&XmlQuery=&File=D%3A%5Czyfiles%5CIndex%20Data%5C00thru05%5CTxt%5C00000015%5CP1001B6V.txt&User=ANONYMOUS&Password=anonymous&SortMethod=h%7C-&MaximumDocuments=1&FuzzyDegree=0&ImageQuality=r75g8/r75g8/x150y150g16/i425&Display=hpfr&DefSeekPage=x&SearchBack=ZyActionL&Back=ZyActionS&BackDesc=Results%20page&MaximumPages=1&ZyEntry=1&SeekPage=x&ZyPURL

Farrugia, N., & Gallina, A. (2008). *Developing Indicators of Territorial Cohesion*. University - Universitetsvej. https://rucforsk.ruc.dk/ws/portalfiles/portal/4339580/Research_Report_1_2008_Farrugia_Gallina.pdf

Fletcher, T. D., Shuster, W., Hunt, W. F., Ashley, R., Butler, D., Arthur, S., Trowsdale, S., Barraud, S., Semadeni-Davies, A., Bertrand-Krajewski, J.-L., Mikkelsen, P. S., Rivard, G., Uhl, M., Dagenais, D., & Viklander, M. (2015). SUDS, LID, BMPs, WSUD and more – The evolution and application of terminology surrounding urban drainage. *Urban Water Journal*, *12*(7), 525-542. https://dx.doi.org/10.1080/1573062x.2014.916314

Griscom, B. W., Adams, J., Ellis, P. W., Houghton, R. A., Lomax, G., Miteva, D. A., Schlesinger, W. H., Shoch, D., Siikamäki, J. V., Smith, P., Woodbury, P., Zganjar, C., Blackman, A., Campari, J., Conant, R. T., Delgado, C., Elias, P., Gopalakrishna, T., Hamsik, M. R., . . . Fargione, J. (2017). Natural climate solutions. *Proceedings of the National Academy of Sciences*, *114*(44), 11645-11650. https://doi.org/10.1073/pnas.1710465114

Griscom, B. W., Busch, J., Cook-Patton, S. C., Ellis, P. W., Funk, J., Leavitt, S. M., Lomax, G., Turner, W. R., Chapman, M., Engelmann, J., Gurwick, N. P., Landis, E., Lawrence, D., Malhi, Y., Schindler Murray, L., Navarrete, D., Roe, S., Scull, S., Smith, P., . . . Worthington, T. (2020). National mitigation

potential from natural climate solutions in the tropics. *Philosophical Transactions of the Royal Society B: Biological Sciences*, *375*(1794), 20190126. https://doi.org/10.1098/rstb.2019.0126

Hansen, R., Rall, E., Chapman, E., Rolf, W., & Pauleit, S. (2017). *Urban Green Infrastructure Planning: A Guide for Practitioners*. G. S. Project. https://ign.ku.dk/english/green-surge/rapporter/D5_3_Urban_GIP_-_A_guide_for_practitioners.pdf

RESOLUTION H1: General Guidelines for the Sustainable Management of Forests in Europe, (1993). https://foresteurope.org/wp-content/uploads/2022/01/MC_helsinki_resolutionH1.pdf

Hewett, C. J. M., Wilkinson, M. E., Jonczyk, J., & Quinn, P. F. (2020). Catchment systems engineering: An holistic approach to catchment management. *WIREs Water*, *7*(3), 14. https://doi.org/10.1002/wat2.1417

IUCN. (2012). *The IUCN programme 2013–2016* (IUCN, Ed.). International Union for Conservation of Nature (IUCN). https://www.iucn.org/sites/default/files/2022-05/wcc-5th-003.pdf

Resolution 69 on Defining Nature-based Solutions (WCC-2016-Res-069), 2 (2016). https://portals.iucn.org/library/sites/library/files/resrecfiles/WCC_2016_RES_069_EN.pdf

IUCN WCPA. (2019). PARKS. The International Journal of Protected Areas and Conservation. In (Vol. 25.2). IUCN. https://doi.org/10.2305/IUCN.CH.2019.PARKS-25-2en

Kabisch, N., Frantzeskaki, N., & Hansen, R. (2022). Principles for urban nature-based solutions. *Ambio*. https://doi.org/10.1007/s13280-021-01685-w

Kabisch, N., Frantzeskaki, N., Pauleit, S., Naumann, S., Davis, M., Artmann, M., Haase, D., Knapp, S., Korn, H., Stadler, J., Zaunberger, K., & Bonn, A. (2016). Nature-based solutions to climate change mitigation and adaptation in urban areas: perspectives on indicators, knowledge gaps, barriers, and opportunities for action. *Ecology and Society*, *21*(2). https://doi.org/10.5751/es-08373-210239

Lavorel, S., Colloff, M. J., McIntyre, S., Doherty, M. D., Murphy, H. T., Metcalfe, D. J., Dunlop, M., Williams, R. J., Wise, R. M., & Williams, K. J. (2015). Ecological mechanisms underpinning climate adaptation services. *Global Change Biology*, *21*(1), 12-31. https://doi.org/10.1111/gcb.12689

Maes, J., & Jacobs, S. (2015). Nature-Based Solutions for Europe's Sustainable Development. *Conservation Letters*, *10*(1), 121-124. https://doi.org/10.1111/conl.12216

Mansourian, S., & Parrotta, J. (2008). *Forest Landscape Restoration - Integrated Approaches to Support Effective Implementation* (S. Mansourian & J. Parrotta, Eds. 1st Edition ed.). Routledge. https://doi.org/10.4324/9781315111872

Sudmeier-Rieux, K., Nehren, U., Sandholz, S., & Doswald, N. (2019). *Disasters and Ecosystems, Resilience in a Changing Climate - Source Book*. United Nations Environment Programme (UNEP). https://doi.org/10.5281/zenodo.3493377

UNEP-WCMC. (2019). *Clarifying terms used in "area-based conservation": - A contribution to the post-2020 global biodiversity framework*. UN Environment Programme World Conservation Monitoring Centre (UNEP-WCMC). https://resources.unep-wcmc.org/products/WCMC_RT235

UNEP. (2022). Resolution adopted by the United Nations Environment Assembly on nature-based solutions for supporting sustainable development. *EA.5/Res.5* 3. https://www.unep.org/resources/resolutions-treaties-and-decisions/UN-Environment-Assembly-5-2

UNEP/CBD. (2000). *Ecosystem approach. Decisions adopted by the Conference of the Parties to the Convention on Biological Diversity at its Fifth Meeting, Nairobi, 15–26 May 2000*. Convention on Biological Diversity. https://www.cbd.int/decision/cop/?id=7148

Wilkie, M. L., Holmgren, P., & Castañeda, F. (2003). *Sustainable Forest Management and the Ecosystem Approach: Two Concepts, One Goal*. https://www.fao.org/3/j1244e/j1244e00.htm

Wong, T. H. F. (2006). Water sensitive urban design - the journey thus far. *Australasian Journal of Water Resources*, *10*(3), 213-222. https://doi.org/10.1080/13241583.2006.11465296

Woodhouse, E., Homewood, K. M., Beauchamp, E., Clements, T., McCabe, J. T., Wilkie, D., & Milner-Gulland, E. J. (2015). Guiding principles for evaluating the impacts of conservation interventions on human well-being. *Philosophical Transactions of the Royal Society B: Biological Sciences*, *370*(1681), 20150103. https://doi.org/10.1098/rstb.2015.0103

WWF. (2020a). *Enhancing NDCs through nature-based solutions - 8 simple recommendations for integrating nature into NDCs*. W. W. F. f. N. (WWF). https://www.worldwildlife.org/publications/enhancing-ndcs-through-nature-based-solutions

WWF. (2020b). *Nature-based solutions for climate change*. W. W. F. f. N. (WWF). https://wwfint.awsassets.panda.org/downloads/wwf_nature_based_solutions_for_climate_change___july_2020_final.pdf
